# Racial/ethnic disparities in colorectal cancer treatment utilization and phase-specific costs, 2000-2014

**DOI:** 10.1371/journal.pone.0231599

**Published:** 2020-04-14

**Authors:** Angela C. Tramontano, Yufan Chen, Tina R. Watson, Andrew Eckel, Chin Hur, Chung Yin Kong

**Affiliations:** 1 Institute for Technology Assessment, Massachusetts General Hospital, Boston, Massachusetts, United States of America; 2 Columbia University Medical Center, New York City, New York, United States of America; 3 Harvard Medical School, Boston, Massachusetts, United States of America; London School of Hygiene and Tropical Medicine, UNITED KINGDOM

## Abstract

**Background:**

Our study analyzed disparities in utilization and phase-specific costs of care among older colorectal cancer patients in the United States. We also estimated the phase-specific costs by cancer type, stage at diagnosis, and treatment modality.

**Methods:**

We used the Surveillance, Epidemiology, and End Results (SEER)-Medicare database to identify patients aged 66 or older diagnosed with colon or rectal cancer between 2000–2013, with follow-up to death or December 31, 2014. We divided the patient’s experience into separate phases of care: staging or surgery, initial, continuing, and terminal. We calculated total, cancer-attributable, and patient-liability costs. We fit logistic regression models to determine predictors of treatment receipt and fit linear regression models to determine relative costs. All costs are reported in 2019 US dollars.

**Results:**

Our cohort included 90,023 colon cancer patients and 25,581 rectal cancer patients. After controlling for patient and clinical characteristics, Non-Hispanic Blacks were less likely to receive treatment but were more likely to have higher cancer-attributable costs within different phases of care. Overall, in both the colon and rectal cancer cohorts, mean monthly cost estimates were highest in the terminal phase, next highest in the staging phase, decreased in the initial phase, and were lowest in the continuing phase.

**Conclusions:**

Racial/ethnic disparities in treatment utilization and costs persist among colorectal cancer patients. Additionally, colorectal cancer costs are substantial and vary widely among stages and treatment modalities. This study provides information regarding cost and treatment disparities that can be used to guide clinical interventions and future resource allocation to reduce colorectal cancer burden.

## Introduction

Colorectal cancer (CRC) is the third most common cancer among both men and women in the United States and the third leading cause of cancer death. Approximately 145,600 new cases and 51,020 deaths are expected in 2019. [1] Survival rates have been increasing over time, with the five-year survival rate currently around 64%. [2] CRC nevertheless remains financially burdensome, with an estimated annual cost exceeding $17 billion, [3] and has long been known to disproportionately afflict certain racial and ethnic minority groups in the U.S. [4–6]

A particularly pronounced and long-standing trend in the U.S. concerns the lower colorectal cancer survival rate among Black patients as compared to White patients. Disparate cancer outcomes can result in part from measurable inequities in screening rates and access to other early detection measures, as well as differences in treatment. [7–9] Indeed, Black colorectal cancer patients are more likely to be diagnosed at later stages and less likely to have potentially curative treatment such as surgery. [4–6, 10] Multiple studies have shown, however, that previously investigated, measurable factors such as stage at diagnosis and treatment receipt are not solely responsible for the observed survival difference between Blacks and Whites in the U.S. [5, 11–13] A recent study suggests that the stage disparity may be abating while incidence and mortality remains disproportionately high among Black patients, underscoring the need for further research into drivers of the survival disparity. [14] The extent to which these trends translate into cancer-associated cost disparities is unknown.

These racial/ethnic disparities in colorectal cancer outcomes pose challenges to health equity among Americans. In a previous study, we determined lung cancer-related costs in the last month of life for White, Black, Asian and Hispanic patients separately and identified significant racial/ethnic cost disparities. [15] Given the known differences in colorectal cancer treatment and outcomes by race/ethnicity, we were interested in seeing whether cost disparities persisted in this context. To pinpoint where disparities might be most pronounced throughout the entire course of care, we used a large, population-based database to estimate CRC care costs by treatment phase. We determined whether cost differences existed between racial/ethnic groups within each phase. We also assessed differences in treatment receipt by race/ethnicity after controlling for potential confounders. An additional focus of our study was to use recent data to update the currently available phase-specific cost estimates for CRC, irrespective of race/ethnicity. [16, 17] These accurate cost estimates are needed in cost-effectiveness analyses evaluating the relative benefits of investing in new treatments. Detailed estimates may also be used to calculate future total costs of CRC care when new screening guidelines or treatment modalities change treatment patterns.

## Materials and methods

### Cohort inclusion and exclusion

We calculated mean (95% CI) monthly costs of colorectal cancer by phase of care using the Surveillance, Epidemiology and End Results (SEER)-Medicare database. SEER contains demographic and clinical information on cancer patients collected from 17 United States registries representing about 28% of the population. [18] The Medicare dataset includes health insurance enrollment information and detailed claims for 97% of the population aged 65 and older. [19] SEER-Medicare is a linkage of these two datasets and includes approximately 95% of the SEER registry population aged 65 and older. [19]

We included patients aged 66 and older who were diagnosed with colorectal cancer as their first and only cancer from 2000–2013 and were continuously enrolled in Medicare Parts A and B coverage from 15 months prior to diagnosis until death or December 31, 2014. We excluded patients enrolled in a Health Maintenance Organization (HMO) during this period because these claims are not available in the SEER-Medicare database. This was to ensure that we had complete claims information for care for all patients. We also excluded patients whose Medicare enrollment was not due to age, who were diagnosed at autopsy only, or who had an unknown cancer stage. Finally, we excluded patients who had an unknown month of cancer diagnosis, a date of death recorded in the Medicare database that differed from that recorded in the SEER database by more than three months, no costs recorded post-diagnosis, costs for claims with unknown dates, or any costs post-death to reduce the potential for including incorrect claims or those due to data entry errors. [20] An inclusion and exclusion criteria flowchart is reported in Fig A S1 File.

### Cost-estimation methods

We based our cost estimation methods on previous studies. [20–23] We defined treatment modalities for patients with stages I-III CRC based on treatment(s) initiated two months prior to cancer diagnosis through six months after diagnosis. The two months prior to diagnosis were included to account for any treatment given to a symptomatic cancer patient who had not yet been diagnosed, as well as possible errors in dates recorded in the claims. Treatment modalities for stage IV CRC patients were defined as treatment(s) ever received. Patients were defined as having treatment if there was a claim in any of the SEER-Medicare claims files with a code pertaining to that treatment. A full list of codes used to define treatment modalities is in Table A S1 File. [24, 25] Patients who had no treatment claims were considered to be not actively treated with surgery, chemotherapy, or radiation and were defined as having received best supportive care. Those who only had a surgery date within the two months prior to diagnosis were not counted as having received surgery. Patients remained in their treatment modality subgroup throughout the analysis.

**Fig 1:**
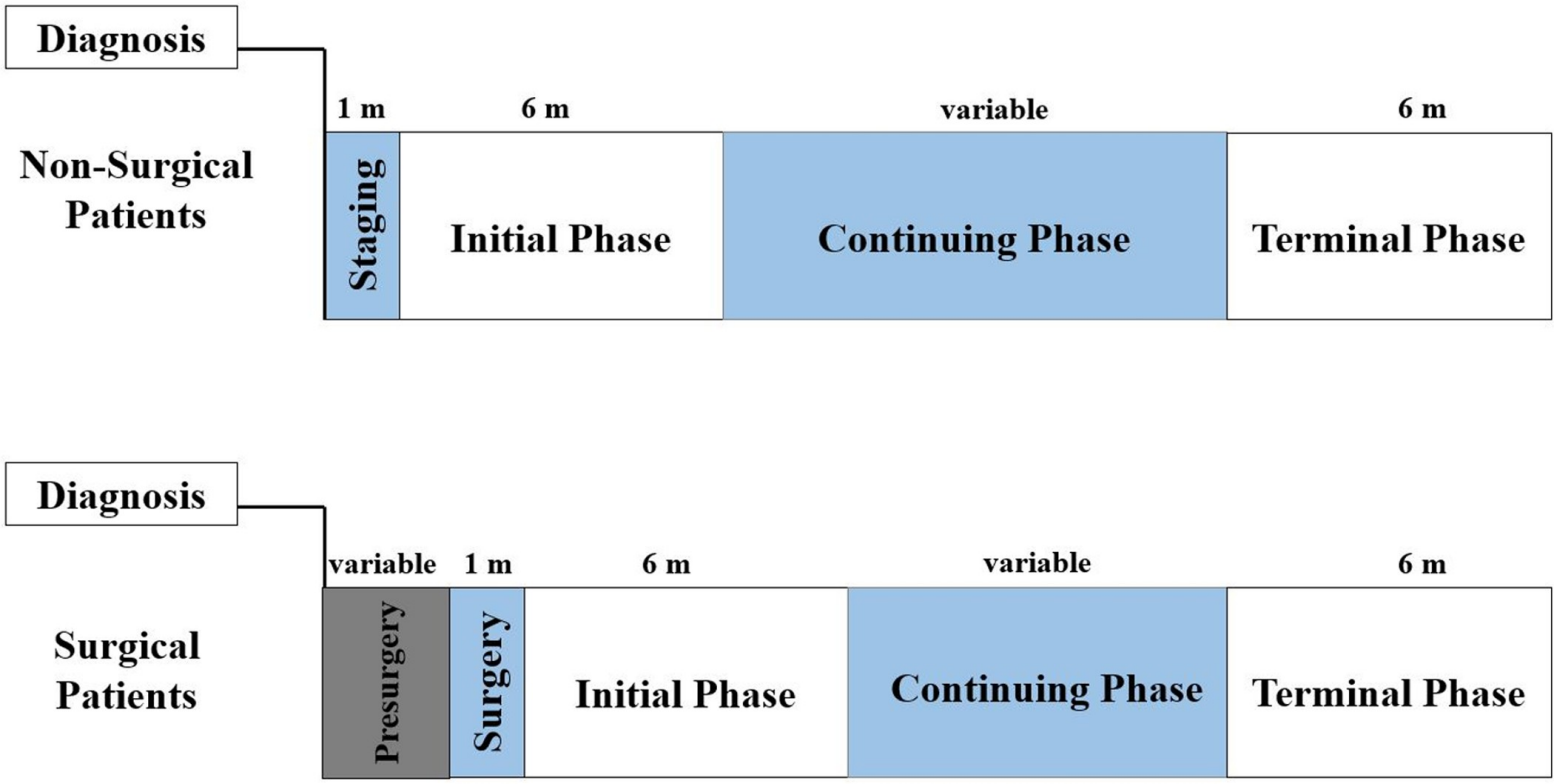
Phases of care timeline for CRC patients.

Each patient’s costs were divided into separate phases of care—staging (or surgery), initial, continuing, and terminal (Fig 1). [16, 20, 21, 23] The staging phase was defined as a one-month stage beginning on the date of diagnosis for nonsurgical patients. Patients who received surgery were given a one-month surgery phase, beginning on the date of major surgery, to allow us to isolate the cost of surgery from any post-operative care. [20] In addition, surgical patients were given a variable presurgery phase beginning on the date of diagnosis. Patients had an initial phase with a maximum length of six months starting after the staging or surgery phase, followed by a continuing phase varying in length between patients depending on survival time. Those patients who died on or before December 31, 2014 had a six-month terminal phase that ended on the date of death. We chose to definite the initial and terminal phases as six months to allow for a more accurate cost estimation of these distinct phases. [20, 23] We allocated costs first to the terminal phase (when applicable), then the staging or surgery phase, the initial phase and, lastly, the continuing phase. For example, a patient who lived 16 months would have a six-month terminal phase, one-month staging phase, six-month initial phase, and three-month continuing phase. CRC patients were only factored into cost averages for phases for which they had at least one month.

### Matched control cohort

We created a control subject cohort from the random sample of 5% of all Medicare enrollees aged 65 years and older who were continuously enrolled in Medicare Part A and B through the study period, were not enrolled in an HMO, and had no cancer diagnosis. This control cohort was matched to CRC cancer patients on an individual (1:1) level within each phase by 5-year age group, sex, and SEER registry region (Northeast, South, Midwest, West). [21]

Because control subjects did not have cancer, each was randomly assigned a “pseudodiagnosis” date matching the cancer diagnosis date of a randomly chosen CRC patient. [21] Care costs for control subjects were allocated to either the continuing or the terminal phase. The terminal phase was defined as the subject’s last six months of life, and the continuing phase was defined as all months between the “pseudodiagnosis” date and the start of the terminal phase. In addition to being matched by 5-year age group, sex, and SEER registry region, cancer patients and control subjects were also matched by phase of care. To determine cancer-attributable costs during both the initial and the continuing phase, patients were matched to control subjects in the continuing phase of care. To determine cancer-attributable costs in the terminal phase, cancer patients who died of their cancer were matched to the control subjects in the continuing phase, while cancer patients who died from other causes were matched to control subjects in the terminal phase. [21] This is to account for the fact that end-of-life care costs are typically high regardless of the cause of death. [16, 26] Cancer-attributable costs were not calculated for the staging, presurgery, or surgery phases.

### Cost analysis

We calculated costs as the sum of Medicare reimbursements, co-insurance reimbursements, and any co-payments and deductibles billed to patients. [21] We calculated total and patient-liability costs for each patient, which may include costs paid by a purchased Medigap policy to help cover a patient’s coinsurance, copayment, and deductible costs. [20, 27] Cancer-attributable costs were estimated by subtracting the matched noncancer subject’s mean monthly phase costs from the cancer patient’s mean monthly phase costs for the initial, continuing, and terminal phases. We report treatment modality costs if at least 10% of patients within a stage received that treatment, except for best supportive care, which is reported for all stage and phase subgroups.

Costs were converted to constant 2019 U.S. dollars by adjusting Part A claims using the CMS Prospective Payment System Hospital Price Index and Part B claims using the Medicare economic index. [28, 29] All costs are reported in 2019 U.S. dollars.

### Statistical analysis

#### Logistic regressions

We fit logistic regression models to determine predictors of treatment receipt (surgery, radiation, or chemotherapy) for colon and rectal cancer. We included the following variables in the models: sex, age at diagnosis, race/ethnicity, marital status, ecological socioeconomic status (SES) quintile, SEER region, urban/rural status, year of diagnosis, AJCC stage at diagnosis, and Charlson comorbidity score (0, 1, 2+). We defined patient race/ethnicity as Non-Hispanic (NH) White (White), NH Black (Black), Hispanic, NH Asian including Pacific Islander (Asian), and “Other” (defined as NH Native American, Native Alaskan, Other, or Unknown in SEER-Medicare). Since the SEER-Medicare database does not include income data at the individual level, we created an ecological SES index using quintiles of ZIP code-level median household incomes from the provided U.S. census data. [30] We categorized SEER regions into Northeast, South, Midwest, and West according to the U.S. Census Bureau’s definition. [31] Comorbidity scores were calculated using the Deyo adaptation of the Charlson comorbidity index for Medicare claims during the year prior to cancer diagnosis. [32–34]

#### Linear regressions

We constructed linear regression models for each phase to examine the association between log-transformed costs and the above patient and clinical characteristics. We used the log-transformed total costs for the staging and surgery phase and the log-transformed cancer-attributable costs for the initial, continuing, and terminal phases. We included treatment modality as an additional covariate. Results are reported as relative cost ratios compared to the reference group.

Statistical significance was defined as *P* < 0.05 in a two‐sided test. We performed all statistical analyses using SAS software, version 9.4 (SAS Institute, Inc., Cary, NC).

### Ethics statement

This study was approved by the Institutional Review Board at Massachusetts General Hospital. The SEER-Medicare data is a limited data set without personal identifiers. The Institutional Review Board waived the requirement for individual informed consent for use of this data.

## Results

### Patient cohort characteristics

Our cohort included 90,023 colon cancer patients and 25,581 rectal cancer patients; their characteristics are reported in Table 1. Among colon cancer patients, 43.1% were male, 82.6% were White, and 19.7% were diagnosed with stage IV disease. The median (25^th^, 75^th^ percentile) age at diagnosis was 78 (73, 84). Among rectal cancer patients, 51.2% were male, 82.3% were White, 33.0%, 17.9% were diagnosed with stage IV disease, and the median (25^th^, 75^th^ percentile) age of diagnosis was 76 (71, 82).

**Table 1:** Description of colorectal cancer patients.

Characteristic	Colon Cancer N (%)	Rectal Cancer N (%)
**Race/Ethnicity**		
White	74,314 (82.6%)	21,051 (82.3%)
Black	7,610 (8.5%)	1,704 (6.7%)
Hispanic	4,136 (4.6%)	1,423 (5.6%)
Asian	3,574 (4.0%)	1,280 (5.0%)
Other	389 (0.4%)	123 (0.5%)
**Sex**		
Male	38,777 (43.1%)	13,108 (51.2%)
Female	51,246 (56.9%)	12,473 (48.8%)
**Age at diagnosis**		
66–69 years	11,366 (12.7%)	4,329 (16.9%)
70–74 years	18,349 (20.4%)	6,132 (24.0%)
75–79 years	20,604 (22.9%)	6,032 (23.6%)
80–84 years	19,690 (21.9%)	4,931 (19.3%)
85+ years	20,014 (22.2%)	4,157 (16.2%)
**Marital Status**		
Married	42,162 (46.8%)	12,651 (49.5%)
Unmarried	43,977 (48.8%)	11,820 (46.2%)
Unknown	3,884 (4.3%)	1,110 (4.3%)
**SES (quintile)**		
0 (lowest)	18,208 (20.2%)	5,167 (20.2%)
1	17,764 (19.7%)	5,273 (20.6%)
2	17,972 (20.0%)	5,106 (20.0%)
3	17,901 (19.9%)	5,144 (20.1%)
4 (highest)	18,178 (20.2%)	4,491 (19.1%)
**SEER Region**		
Northeast	20,714 (23.0%)	5,983 (23.4%)
South	22,969 (25.5%)	6,466 (25.3%)
Midwest	13,238 (14,7%)	3,491 (13.7%)
West	33,192 (36.8%)	9,641 (39.7%)
**Metro**		
Metro/urban	78,765 (87.5%)	22,351 (87.4%)
Less urban/rural	11,258 (12.5%)	3,230 (12.6%)
**Year of diagnosis**		
2000–2004	37,612 (41.8%)	11,074 (43.3%)
2005–2009	31,574 (35.1%)	8,877 (34.7%)
2010–2013	20,837 (23.2%)	5,630 (22.0%)
**Stage at diagnosis**		
Stage I	21,036 (23.4%)	8,333 (32.6%)
Stage II	28,387 (31.5%)	6,303 (24.6%)
Stage III	22,903 (25.4%)	6,357 (24.9%)
Stage IV	17,697 (19.7%)	4,588 (17.9%)
**Charlson comorbidity score**		
0	36,265 (40.3%)	12,299 (48.1%)
1	24,972 (27.7%)	6,934 (27.1%)
2+	28,786 (32.0%)	6,348 (24.8%)
**Treatment modality**		
Best supportive care	7,894 (8.8%)	2,679 (10.5%)
Surgery	58,799 (65.3%)	10,318 (40.3%)
Surgery and Radiation	653 (0.7%)	2,106 (8.2%)
Surgery and Chemotherapy	18,876 (20.9%)	2,183 (8.5%)
Surgery and Chemoradiation	1,539 (1.7%)	5,016 (19.6%)
Radiation	176 (0.2%)	1,062 (4.5%)
Chemotherapy	1,840 (2.0%)	574 (2.2%)
Chemoradiation	246 (0.3%)	1,543 (6.0%)
**Cause of death**		
Colorectal cancer	26,892 (29.9%)	9,085 (35.5%)
Operative‡	5,329 (6.0%)	870 (3.4%)
All other causes	29,006 (32.2%)	7,679 (30.0%)
Alive	28,796 (32.0%)	7,947 (31.1%)

‡ Operative death is defined as death within 30 days of surgery to remove colorectal cancer.

Among treatment modalities, surgery alone was the most common, which was received by 65.3% of colon cancer patients and 40.3% of rectal cancer patients. Approximately 8.8% of colon cancer patients and 10.5% of rectal cancer patients received best supportive care. By the end of the study period, 29.9% colon cancer patients and 35.5% rectal cancer patients had died of their disease.

### Racial/ethnic disparities in treatment receipt

We considered whether there were racial/ethnic differences in treatment receipt after controlling for known patient and clinical characteristics. As shown in Table 2, treatment receipt was significantly lower for Blacks compared to Whites, after controlling for other covariates. Specifically, Black patients were less likely to receive surgery (OR: 0.76; 95% CI: 0.62–0.72; p<0.0001), radiation (OR: 0.76; 95% CI: 0.65–0.89; p = 0.0005), or chemotherapy (OR: 0.798; 95% CI: 0.74–0.84; p<0.0001) when compared to White patients.

**Table 2:** Characteristics associated with colon cancer treatment.

	Surgery Receipt (N = 79,867)		Radiation Receipt (N = 2,614)		Chemotherapy Receipt (N = 22,501)	
Characteristic	OR (95% CI)	p-value	OR (95% CI)	p-value	OR (95% CI)	p-value
**Race/Ethnicity (ref = White)**						
Black	0.67 (0.62–0.72)	<0.0001	0.76 (0.65–0.89)	0.0005	0.79 (0.74–0.84)	<0.0001
Hispanic	0.89 (0.81–0.995)	0.04	0.97 (0.81–1.17)	0.75	1.08 (0.99–1.18)	0.07
Asian	1.00 (0.88–1.13)	0.99	0.90 (0.72–1.11)	0.34	1.14 (1.04–1.25)	0.006
Other	0.39 (0.29–0.52)	<0.0001	0.69 (0.34–1.41)	0.31	0.71 (0.53–0.96)	0.02
**Sex (ref = Male)**						
Female	1.41 (1.34–1.48)	<0.0001	0.92 (0.85–1.00)	0.051	1.01 (0.97–1.05)	0.74
**Age at diagnosis (ref = 66–69)**						
70–74	0.95 (0.87–1.02)	0.22	0.79 (0.71–0.89)	<0.0001	0.72 (0.68–0.76)	<0.0001
75–79	0.87 (0.79–0.94)	0.0008	0.63 (0.56–0.70)	<0.0001	0.47 (0.44–0.50)	<0.0001
80–84	0.73 (0.67–0.79)	<0.0001	0.45 (0.40–0.52)	<0.0001	0.21 (0.20–0.22)	<0.0001
85+	0.48 (0.44–0.52)	<0.0001	0.26 (0.23–0.31)	<0.0001	0.07 (0.06–0.07)	<0.0001
**Marital Status (ref = Married)**						
Unmarried	0.76 (0.72–0.80)	<0.0001	0.83 (0.76–0.91)	0.0003	0.64 (0.62–0.67)	<0.0001
Unknown	0.55 (0.50–0.62)	<0.0001	0.89 (0.72–1.10)	0.27	0.74 (0.67–0.81)	<0.0001
**SES (ref = 0)**						
1	1.054 (0.97–1.12)	0.22	0.99 (0.87–1.12)	0.86	1.05 (0.99–1.11)	0.11
2	1.04 (0.97–1.12)	0.25	1.04 (0.92–1.18)	0.54	1.13 (1.06–1.20)	<0.0001
3	1.11 (1.03–1.20)	0.005	0.97 (0.85–1.11)	0.64	1.15 (1.08–1.22)	<0.0001
4 (highest)	1.14 (1.05–1.23)	0.001	1.04 (0.91–1.18)	0.54	1.19 (1.13–1.28)	<0.0001
**SEER Region (ref = Northeast)**						
South	1.01 (0.94–1.09)	0.70	0.89 (0.79–1.00)	0.06	0.83 (0.79–0.88)	<0.0001
Midwest	0.97 (0.90–1.05)	0.51	0.82 (0.71–0.95)	0.006	0.87 (0.80–0.91)	<0.0001
West/Hawaii	0.93 (0.88–0.99)	0.03	0.94 (0.85–1.05)	0.26	0.86 (0.81–0.90)	<0.0001
**Residence (ref = Metro/Urban)**						
Less Urban/Rural	0.93 (0.86–1.00)	0.051	0.98 (0.86–1.12)	0.77	0.95 (0.89–1.01)	0.10
**Year of Diagnosis (ref = 2000–2004)**						
2005–2009	0.88 (0.83–0.92)	<0.0001	0.96 (0.88–1.05)	0.39	0.87 (0.83–0.91)	<0.0001
2010–2013	0.69 (0.64–0.72)	<0.0001	0.89 (0.81–0.99)	0.04	0.85 (0.81–0.89)	<0.0001
**AJCC Stage (ref = I)**						
II	3.37 (3.11–3.64)	<0.0001	2.41 (2.03–2.87)	<0.0001	7.40 (6.69–8.07)	<0.0001
III	4.93 (4.47–5.43)	<0.0001	2.42 (2.03–2.89)	<0.0001	50.76 (46.22–55.74)	<0.0001
IV	0.23 (0.21–0.24)	<0.0001	11.34 (9.68–13.28)	<0.0001	45.97 (41.80–50.56)	<0.0001
**Charlson Score (ref = 0)**						
1	1.12 (1.06–1.19)	<0.0001	0.88 (0.79–0.89)	0.006	0.81 (0.77–0.84)	<0.0001
2+	0.96 (0.91–1.01)	0.14	0.71 (0.96–0.78)	<0.0001	0.49 (0.47–0.52)	<0.0001

Significant differences were also observed in other racial/ethnic subgroups. Hispanic patients were less likely to receive surgery than Whites (OR: 0.89; 95% CI: 0.81–0.995; p = 0.04), and Asian patients were more likely to receive chemotherapy when compared to Whites (OR: 1.14; 95% CI: 1.04–1.25; p = 0.006). Compared to Whites, patients listed with “Other” as their race/ethnicity were less likely to receive surgery (OR: 0.39; 95% CI: 0.29–0.52; p<0.0001) or chemotherapy (OR: 0.71; 95% CI: 0.53–0.96; p = 0.02).

Significant differences in rectal cancer treatment receipt were also observed and are reported in Table 3. Blacks (OR: 0.64; 95% CI: 0.57–0.72; p<0.0001), Hispanics (OR: 0.82; 95% CI: 0.71–0.95; p = 0.006), and patients listed as “Other” (OR: 0.43; 95% CI: 0.29–0.63; p<0.0001) were less likely than Whites to receive surgery. Asians were less likely to receive radiation when compared to Whites (OR 0.85; 95% CI: 0.58–0.96; p = 0.01). Compared to Whites, Blacks (OR: 0.769; 95% CI: 0.67–0.86; p = 0.0002), Asians (OR: 0.84; 95% CI: 0.73–0.96; p = 0.02), and patients listed as “Other” (OR: 0.60; 95% CI: 0.38–0.93; p = 0.02) were less likely to receive chemotherapy, while Hispanics were more likely to receive chemotherapy (OR: 1.23; 95% CI: 1.08–1.39; p = 0.001).

**Table 3:** Characteristics associated with rectal cancer treatment.

	Surgery Receipt (N = 19,623)		Radiation Receipt (N = 9,727)		Chemotherapy Receipt (N = 9,316)	
Characteristic	OR (95% CI)	p-value	OR (95% CI)	p-value	OR (95% CI)	p-value
**Race/Ethnicity (ref = White)**						
Black	0.64 (0.57–0.72)	<0.0001	0.98 (0.87–1.09)	0.66	0.76 (0.67–0.86)	0.0002
Hispanic	0.82 (0.71–0.95)	0.006	1.11 (0.98–1.24)	0.10	1.23 (1.08–1.39)	0.002
Asian	1.13 (0.97–1.32)	0.13	0.85 (0.75–0.96)	0.01	0.84 (0.73–0.96)	0.01
Other	0.43 (0.29–0.63)	<0.0001	0.86 (0.58–1.28)	0.46	0.60 (0.38–0.93)	0.02
Sex (ref = Male)						
Female	1.20 (1.12–1.28)	<0.0001	0.94 (0.89–0.999)	0.047	0.96 (0.90–1.02)	0.18
**Age at diagnosis (ref = 66–69)**						
70–74	0.99 (0.90–1.10)	0.90	0.86 (0.79–0.94)	0.003	0.79 (0.73–0.87)	<0.0001
75–79	0.90 (0.81–1.00)	0.045	0.71 (0.65–0.77)	<0.0001	0.55 (0.50–0.59)	<0.0001
80–84	0.77 (0.69–0.85)	<0.0001	0.54 (0.49–0.58)	<0.0001	0.32 (0.29–0.35)	<0.0001
85+	0.52 (0.47–0.58)	<0.0001	0.34 (0.31–0.37)	<0.0001	0.12 (0.11–0.14)	<0.0001
**Marital Status (ref = Married)**						
Unmarried	0.74 (0.69–0.79)	<0.0001	0.90 (0.85–0.96)	0.0005	0.76 (0.71–0.81)	<0.0001
Unknown	0.61 (0.53–0.71)	<0.0001	0.72 (0.63–0.83)	<0.0001	0.78 (0.67–0.91)	0.002
**SES (ref = 0)**						
1	0.96 (0.78–1.06)	0.39	1.01 (0.92–1.09)	0.96	1.04 (0.94–1.14)	0.47
2	0.99 (0.90–1.02)	0.91	1.04 (0.96–1.13)	0.37	1.17 (1.06–1.28)	0.001
3	0.97 (0.88–1.08)	0.66	1.02 (0.93–1.11)	0.68	1.13 (1.03–1.25)	0.009
4 (highest)	1.03 (0.93–1.15)	0.55	1.06 (0.97–1.16)	0.17	1.18 (1.07–1.30)	0.001
**SEER Region (ref = Northeast)**						
South	1.08 (0.98–1.19)	0.12	1.02 (0.95–1.11)	0.55	0.94 (0.86–1.02)	0.18
Midwest	1.15 (1.03–1.28)	0.02	0.96 (0.88–1.05)	0.41	0.98 (0.89–1.08)	0.75
West/Hawaii	1.07 (0.98–1.16)	0.13	0.95 (0.88–1.02)	0.17	0.82 (0.76–0.89)	0.0007
**Residence (ref = Metro/Urban)**						
Less Urban/Rural	0.96 (0.85–1.06)	0.40	0.95 (0.87–1.02)	0.26	0.99 (0.90–1.08)	0.83
**Year of Diagnosis (ref = 2000–2004)**						
2005–2009	0.69 (0.64–0.74)	<0.0001	1.23 (1.16–1.32)	0.01	0.92 (0.86–0.99)	0.02
2010–2013	0.41 (0.37–0.44)	<0.0001	1.47 (1.37–1.57)	<0.0001	0.82 (0.76–0.89)	<0.0001
**AJCC Stage (ref = I)**						
II	1.60 (1.45–1.73)	<0.0001	3.14 (2.92–3.38)	<0.0001	3.45 (3.18–3.76)	<0.0001
III	2.27 (2.07–2.50)	<0.0001	3.26 (3.03–3.51)	<0.0001	7.21 (6.64–7.84)	<0.0001
IV	0.34 (0.31–0.37)	<0.0001	1.77 (1.63–1.92)	<0.0001	7.64 (6.99–8.36)	<0.0001
**Charlson Score (ref = 0)**						
1	1.15 (1.06–1.24)	0.0006	0.87 (0.82–0.93)	<0.0001	0.88 (0.82–0.94)	0.0002
2+	1.01 (0.94–1.10)	0.72	0.65 (0.61–0.70)	<0.0001	0.56 (0.52–0.60)	<0.0001

### Racial/ethnic disparities in phase-specific costs

To determine whether there were disparities in costs, we analyzed the relative cost ratios within each phase of care after controlling for patient and clinical characteristics. Several significant differences were detected in each phase. Specifically, after controlling for other covariates, including stage at diagnosis, treatment type, SES, urban/rural residence, and region, Black colon cancer patients had significantly higher relative costs when compared to Whites in both the staging (1.19; 95% CI: 1.02–1.40; p = 0.03) and surgery phases (1.11; 95% CI: 1.09–1.13; p<0.0001) (Table B S1 File). Hispanic and Asian patients had significantly higher relative costs when compared to Whites in the surgery phase (Hispanic: 1.02; 95% CI:1.00–1.05; p = 0.046 and Asian: 1.08; 95% CI: 1.06–1.11; p<0.0001), and Other patients had lower costs compared to Whites (0.93; 95% CI 0.87–1.00; p = 0.049).

Relative cost ratios in monthly cancer-attributable costs by initial, continuing, or terminal phase for colon cancer patients are reported in Table 4. Black patients had significantly higher relative costs when compared to White patients in the initial phase (1.15; 95% CI: 1.09–1.22; p<0.0001, the continuing phase (1.27; 95% CI: 1.20–1.35; p<0.0001) and the terminal phase (1.24; 95% CI: 1.19–1.30; p<0.0001). Hispanic and Asian patients had higher relative terminal phase costs as well (Hispanic: 1.09; 95% CI: 1.03–1.16; p = 0.004 and Asian: 1.07; 95% CI: 1.00–1.15; p = 0.04).

**Table 4:** Association between cancer-attributable costs and characteristics by phase of care: Colon cancer.

	Initial Phase			Continuing Phase			Terminal Phase		
Characteristic	Relative cost ratio	95% CI	P-value	Relative cost ratio	95% CI	P-value	Relative cost ratio	95% CI	P-value
Race/Ethnicity ref = White									
Black	1.15	1.09–1.22	<0.0001*	1.27	1.20–1.35	<0.0001*	1.24	1.19–1.30	<0.0001*
Hispanic	1.02	0.95–1.09	0.63	1.13	1.05–1.22	0.001	1.09	1.03–1.16	0.004*
Asian	0.96	0.9–1.03	0.28	1.02	0.94–1.11	0.85	1.07	1.00–1.15	0.04*
Other	0.94	0.76–1.16	0.55	0.91	0.72–1.16	0.16	1.02	0.83–1.25	0.87
Sex ref = Male									
Female	0.95	0.92–0.98	0.001*	0.93	0.90–0.96	<0.0001*	0.97	0.94–0.99	0.02*
Age at Diagnosis ref = 66–69									
70–74	0.95	0.91–0.99	0.02*	0.99	0.94–1.04	0.62	0.93	0.89–0.98	0.004*
75–79	0.90	0.86–0.94	<0.0001*	0.96	0.91–1.01	0.09	0.85	0.81–0.89	<0.0001*
80–84	0.82	0.78–0.86	<0.0001*	0.93	0.89–0.99	0.02*	0.75	0.72–0.79	<0.0001*
85+	0.83	0.79–0.88	<0.0001*	0.97	0.91–1.03	<0.0001*	0.72	0.69–0.76	<0.0001*
Marital Status ref = Unmarried									
Married	1.05	1.01–1.08	0.005*	1.07	1.03–1.11	0.0004*	1.00	0.97–1.03	1.00
Unknown	1.04	0.97–1.12	0.30	0.95	0.87–1.02	0.17	0.97	0.91–1.03	0.34
SES ref = 0									
1	1.01	0.97–1.06	0.69	0.98	0.93–1.03	0.36	0.94	0.91–0.98	0.003*
2	1.00	0.95–1.04	0.91	0.97	0.92–1.02	0.27	0.96	0.93–1.00	0.06
3	1.01	0.96–1.06	0.68	0.99	0.94–1.04	0.62	0.96	0.92–1.00	0.04*
4 highest	1.02	0.97–1.07	0.47	0.95	0.9–1.00	0.04*	0.96	0.92–1.00	0.04*
SEER Region ref = Northeast									
South	0.87	0.84–0.91	<0.0001*	0.94	0.90–0.99	0.01*	0.79	0.76–0.82	<0.0001*
Midwest	0.86	0.82–0.91	<0.0001*	0.91	0.86–0.96	0.001*	0.83	0.8–0.87	<0.0001*
West/Hawaii	0.86	0.83–0.89	<0.0001*	0.96	0.92–1.00	0.04*	0.93	0.9–0.96	<0.0001*
Residence ref = Metro/Urban									
Less Urban/Rural	0.92	0.88–0.96	0.0003*	0.90	0.85–0.96	0.001	0.90	0.86–0.94	<0.0001
Year of Diagnosis ref = 2000–2004									
2005–2009	1.38	1.33–1.42	<0.0001*	1.06	1.02–1.10	0.002*	1.16	1.13–1.19	<0.0001*
2010–2013	1.28	1.24–1.33	<0.0001*	1.06	1.02–1.11	0.003*	1.31	1.26–1.35	<0.0001*
AJCC Stage ref = I									
II	0.92	0.88–0.96	<0.0001*	1.09	1.05–1.14	<0.0001*	1.08	1.04–1.12	0.001*
III	1.16	1.11–1.21	<0.0001*	1.30	1.24–1.37	<0.0001*	1.18	1.13–1.23	<0.0001*
IV	1.72	1.62–1.81	<0.0001*	3.10	2.90–3.31	<0.0001*	1.67	1.60–1.75	<0.0001*
Charlson Score ref = 0									
1	1.07	1.04–1.11	0.001*	1.13	1.09–1.18	<0.0001*	1.10	1.07–1.14	<0.0001*
2+	1.28	1.24–1.33	<0.0001*	1.51	1.45–1.57	<0.0001*	1.35	1.31–1.39	<0.0001*
Treatment ref = BSC									
Surgery	0.88	0.81–0.96	0.002*	0.87	0.79–0.95	0.001*	1.05	1.01–1.10	0.02*
Surgery and Radiation	1.16	0.99–1.35	0.07	0.90	0.73–1.11	0.32	0.98	0.86–1.11	0.73
Surgery and Chemotherapy	1.44	1.32–1.56	<0.0001*	1.03	0.94–1.13	0.52	0.91	0.87–0.95	0.0001*
Surgery and Chemoradiation	1.88	1.68–2.09	<0.0001*	1.28	1.13–1.46	0.0002*	0.85	0.78–0.92	0.0001*
Radiation	1.72	1.12–2.65	0.01*	1.42	0.79–2.90	0.34	1.22	1.00–1.50	0.054
Chemotherapy	1.88	1.67–2.12	0.001*	1.61	1.38–1.87	<0.0001*	0.94	0.87–1.01	0.10
Chemoradiation	1.78	1.43–2.21	<0.0001*	1.64	1.25–2.16	0.0003*	0.92	0.77–1.09	0.32

*P-value significant at <0.05 for multiple linear regression using log transformation of cost.

We conducted a similar relative cost ratio analysis among rectal cancer patients to determine whether racial/ethnic differences in the cost estimates existed in this cancer type. Black patients with rectal cancer had significantly higher relative costs when compared to Whites in the surgery phase (1.08; 95% CI: 1.03–1.14; p0.003), while in the staging phase we found no significant relative cost ratios by race/ethnicity (Table C S1 File).

Relative ratios in monthly cancer-attributable costs by initial, continuing, and terminal phase for rectal cancer patients are reported in Table 5. There were no significant racial/ethnic relative cost ratios reported during the initial phase. In the continuing phase, however, Blacks had a significantly higher relative cost when compared to White patients (1.37; 95% CI: 1.27–1.54; p<0.0001). Asian patients also had a significantly higher cost (1.19; 95% CI: 1.04–1.35; p = 0.01). In the terminal phase, Blacks (1.28; 95% CI: 1.18–1.39; p<0.0001), Hispanics (1.11; 95% CI: 1.01–1.22; p = 0.03), and Asians (1.19; 95% CI: 1.07–1.32; p = 0.001) had significantly higher relative costs when compared to Whites.

**Table 5:** Association between cancer-attributable costs and characteristics by phase of care: Rectal cancer.

	Initial Phase			Continuing Phase			Terminal Phase		
Characteristic	Relative cost ratio	95% CI	P-value	Relative cost ratio	95% CI	P-value	Relative cost ratio	95% CI	P-value
Race/Ethnicity (ref = White)									
Black	1.05	0.97–1.14	0.24	1.37	1.23–1.54	<0.0001*	1.28	1.18–1.39	<0.0001*
Hispanic	0.96	0.88–1.05	0.34	1.04	0.93–1.17	0.47	1.11	1.01–1.22	0.03*
Asian	0.95	0.87–1.04	0.30	1.19	1.04–1.35	0.01	1.19	1.07–1.32	0.001*
Other	0.95	0.72–1.26	0.75	1.08	0.76–1.55	0.66	1.14	0.80–1.61	0.48
Sex (ref = Male)									
Female	1.02	0.98–1.06	0.32	0.96	0.9–1.01	0.12	0.91	0.87–0.95	0.0003*
Age at Diagnosis (ref = 66–69)									
70–74	0.94	0.89–1.00	0.04*	0.95	0.87–1.02	0.15	0.90	0.83–0.96	0.003*
75–79	0.85	0.80–0.90	<0.0001*	0.97	0.89–1.05	0.44	0.81	0.76–0.88	<0.0001*
80–84	0.79	0.74–0.85	<0.0001*	0.90	0.82–0.98	0.02*	0.75	0.69–0.81	<0.0001*
85+	0.72	0.67–0.78	<0.0001*	0.99	0.89–1.10	0.83	0.67	0.62–0.73	<0.0001*
Marital Status (ref = Unmarried)									
Married	1.03	0.99–1.08	0.19	1.08	1.02–1.15	0.007*	0.98	0.94–1.03	0.46
Unknown	1.07	0.97–1.18	0.20	1.10	0.96–1.27	0.16	0.96	0.86–1.07	0.49
SES (ref = 0)									
1	1.06	1.00–1.13	0.06	1.00	0.92–1.09	0.96	0.99	0.92–1.05	0.66
2	1.06	0.99–1.13	0.08	0.99	0.91–1.08	0.89	0.97	0.91–1.04	0.42
3	1.02	0.95–1.09	0.58	1.00	0.92–1.09	0.97	1.00	0.93–1.07	0.93
4 (highest)	1.01	0.95–1.08	0.67	0.92	0.84–1.01	0.08	0.95	0.89–1.02	0.19
SEER Region (ref = Northeast)									
South	0.86	0.81–0.91	<0.0001*	0.86	0.79–0.93	0.0003*	0.84	0.78–0.89	<0.0001*
Midwest	0.91	0.85–0.97	0.007*	0.90	0.82–0.99	0.02*	0.87	0.81–0.94	0.0002*
West/Hawaii	0.99	0.94–1.04	0.61	0.92	0.86–0.99	0.03*	0.94	0.89–1.00	0.04
Residence (ref = Metro/Urban)									
Less Urban/Rural	0.88	0.82–0.94	0.0002*	0.91	0.83–1.00	0.053	0.81	0.75–0.87	<0.0001*
Year of Diagnosis (ref = 2000–2004)									
2005–2009	1.29	1.23–1.35	<0.0001*	1.02	0.96–1.08	0.52	1.17	1.12–1.23	<0.0001*
2010–2013	1.26	1.20–1.33	<0.0001*	1.07	1.00–1.15	0.07	1.34	1.26–1.43	<0.0001*
AJCC Stage (ref = I)									
II	1.18	1.11–1.24	<0.0001*	1.19	1.10–1.28	<0.0001*	1.12	1.05–1.20	0.001*
III	1.31	1.24–1.39	<0.0001*	1.44	1.33–1.55	<0.0001*	1.11	1.04–1.18	0.002*
IV	1.75	1.63–1.88	<0.0001*	3.01	2.72–3.33	<0.0001*	1.46	1.37–1.56	<0.0001*
Charlson Score (ref = 0)									
1	1.07	1.02–1.12	0.003*	1.08	1.01–1.15	0.02*	1.15	1.09–1.21	<0.0001*
2+	1.20	1.14–1.26	<0.0001*	1.37	1.28–1.47	<0.0001*	1.37	1.30–1.44	<0.0001*
Treatment (ref = BSC)									
Surgery	1.23	1.11–1.36	<0.0001*	0.93	0.83–1.05	0.25	0.98	0.91–1.06	0.64
Surgery and Radiation	1.37	1.22–1.53	<0.0001*	0.91	0.79–1.05	0.19	0.89	0.80–0.98	0.02*
Surgery and Chemotherapy	1.72	1.53–1.92	<0.0001*	1.17	1.01–1.35	0.04*	0.82	0.75–0.90	<0.0001*
Surgery and Chemoradiation	1.94	1.74–2.15	<0.0001*	1.02	0.90–1.16	0.77	0.90	0.82–0.98	0.02*
Radiation	2.20	1.94–2.49	<0.0001*	1.31	1.10–1.55	0.002*	0.96	0.86–1.07	0.43
Chemotherapy	2.28	1.93–2.68	<0.0001*	1.50	1.20–1.87	0.004*	0.96	0.85–1.09	0.54
Chemoradiation	3.02	2.69–3.40	<0.0001*	1.41	1.21–1.64	<0.0001*	0.91	0.82–1.00	0.049*

*P-value significant at <0.05 for multiple linear regression using log transformation of cost.

### Detailed phase-specific cost estimates

**Fig 2:**
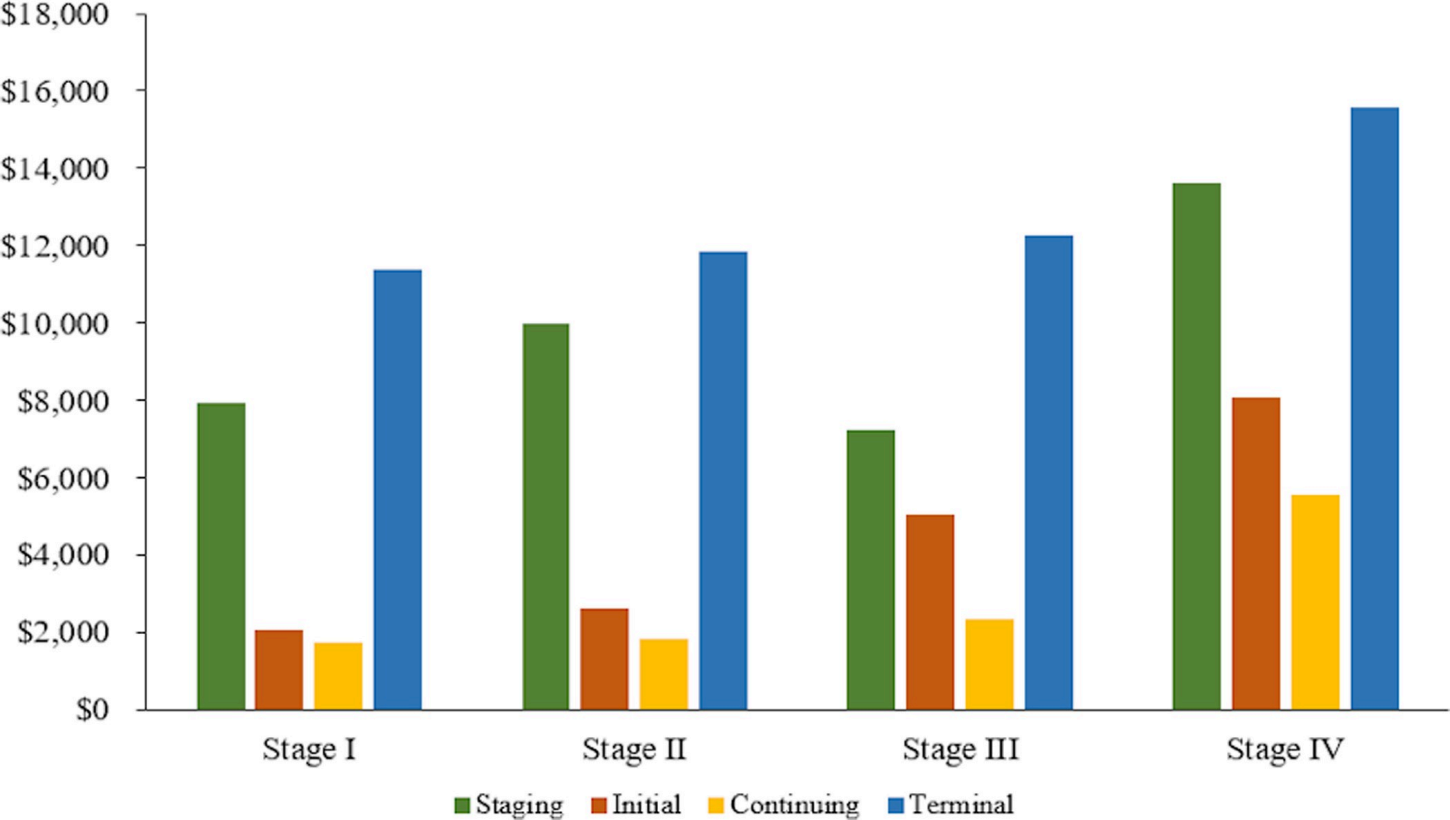
Mean monthly total costs by stage and phase: Colon cancer.

**Fig 3:**
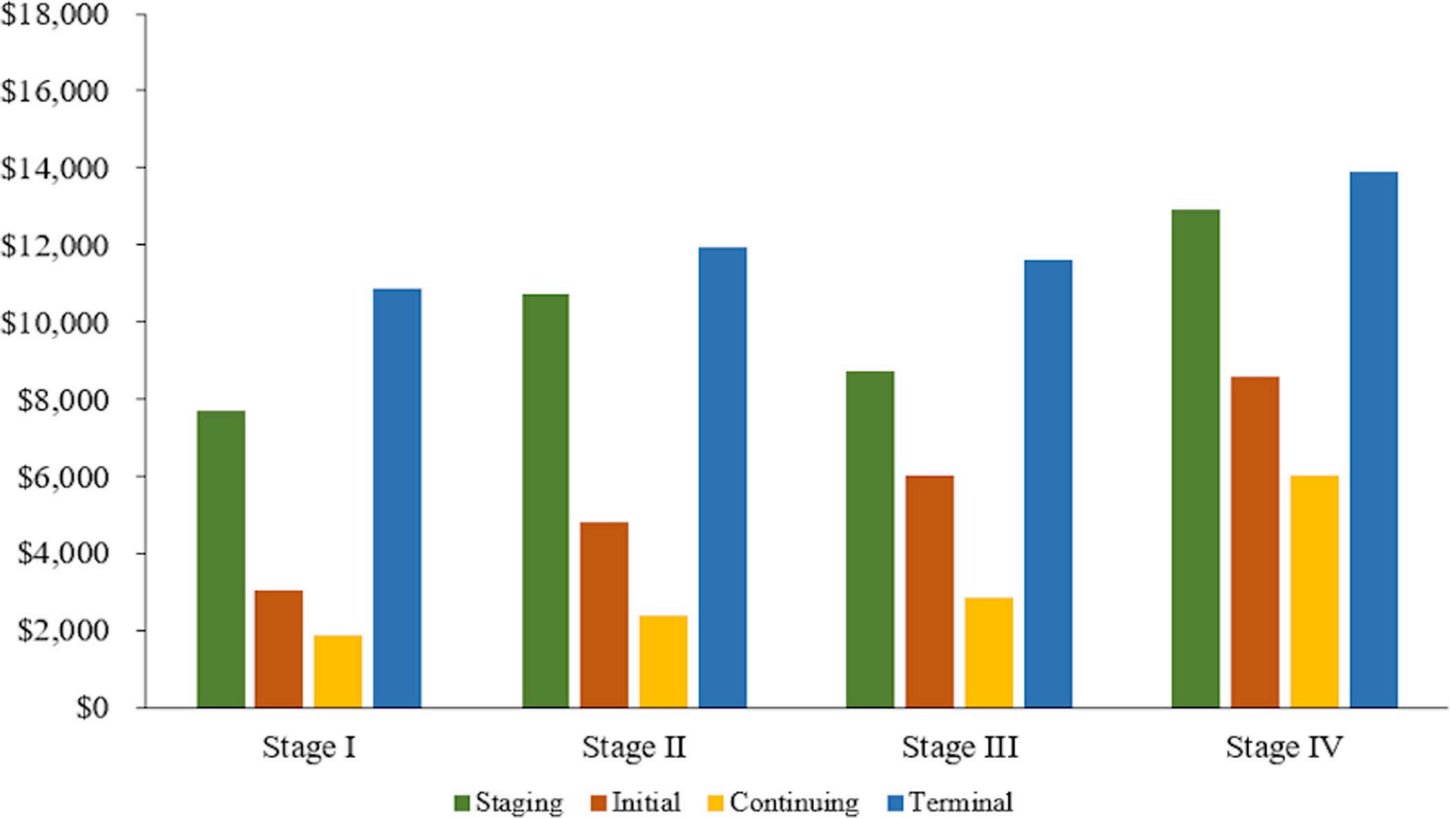
Mean monthly total costs by stage and phase: Rectal cancer.

For comparison among cancer types, stages, and phases of care, we report total, cancer-attributable, and patient-liability cost estimates by stage at diagnosis and treatment phase in Table 6. Additionally, mean (95% CI) monthly total cost estimates for each cancer stage at diagnosis and treatment phase are shown in Fig 2 for colon cancer and Fig 3 for rectal cancer. No matter the stage at diagnosis, total monthly cost estimates were high in the staging phase, decreased in the initial and continuing phases, and increased again in the terminal phase. Among colon cancer patients, those diagnosed with stage IV cancer had the highest total costs in each phase: $13,605 ($12,867-$14,342) in the staging phase, $8,034 ($7,880-$8,190) in the initial phase, $5,541 ($5,392-$5,689) in the continuing phase, and $15,537 ($15,280-$15,793) in the terminal phase. The highest total costs among rectal patients were also among those diagnosed in stage IV: $12,911 ($11,905-$13,916) in the staging phase, $8,536 ($8,239-$8,832) in the initial phase, $5,991 ($5,730-$6,251) in the continuing phase, and $13,857 ($13,436-$14,277) in the terminal phase. The total cost estimates for operative death were $48,718 ($47,905-$49,531) for surgical colon cancer patients and $42,654 ($40,614-$44,694) for surgical rectal cancer patients.

**Table 6:** Mean monthly cost estimates for each phase by AJCC stage at diagnosis.

	Monthly total cost (95% CI)	Monthly Patient-liability cost (95% CI)	Monthly Cancer-attributable cost (95% CI)
**Colon**			
**Stage I**			
Surgery	$32,782 ($32,000-$33,142)	$2,212 ($2,190-$2,240)	*
Staging	$7,894 ($7,155-$8,632)	$959 ($884-$1,033)	*
Initial	$2,046 ($1,980-$2,110)	$243 ($234-$250)	$935 ($873-$997)
Continuing	$1,688 ($1,648-$1,727)	$224 ($217-$227)	$565 ($525-$604)
Terminal	$11,367 ($11,308-$11,695)	$907 ($885-$928)	$5,354 ($5,022-$5,685)
**Stage II**			
Surgery	$38,681 ($38,364-$38,999)	$2,464 ($2,440-$2,490)	*
Staging	$9,968 ($8,560-$11,375)	$877 ($762-$991)	*
Initial	$2,596 ($2,540-$2,660)	$341 ($331-$348)	$1,473 ($1,410-$1,530)
Continuing	$1,818 ($1,779-$1,856)	$240 ($236-$246)	$684 ($645-$722)
Terminal	$11,830 ($11,559-$12,118)	$929 ($910-$947)	$6,776 ($6,491-$7,060)
**Stage III**			
Surgery	$39,961 ($39,607-$40,316)	$2,504 ($2,470-$2,530)	*
Staging	$7,216 ($5,426-$9,005)	$611 ($484-$737)	*
Initial	$4,992 ($4,910-$5,080)	$738 ($720-$746)	$3,886 ($3,800-$3,970)
Continuing	$2,328 ($2,279-$2,376)	$329 ($322-$335)	$1,212 ($1,163-$1,260)
Terminal	$12,234 ($11,963-$12,504)	$990 ($913-$998)	$8,544 ($8,269-$8,818)
**Stage IV**			
Surgery	$41,818 ($41,266-$42,371)	$2,631 ($2,580-$2,680)	*
Staging	$13,605 ($12,867-$14,342)	$1,559 ($1,489-$1,628)	*
Initial	$8,034 ($7,880-$8,190)	$1,234 $1,195-$1,247)	$6,959 ($6,800-$7,120)
Continuing	$5,541 ($5,392-$5,689)	$889 ($858-$906)	$4,465 ($4,315-$4,614)
Terminal	$15,537 ($15,280-$15,793)	$1,258 ($1,226-$1,260)	$13,316 ($13,058-$13,573)
**Rectal**			
**Stage I**			
Surgery	$27,284 ($26,583-$27,984)	$2,160 ($2,115-$2,204)	*
Staging	$7,671 ($6,992-$8,349)	$1,064 ($987-$1,140)	*
Initial	$2,998 ($2,889-$3,106)	$394 ($375-$407)	$1,890 ($1,781-$1,998)
Continuing	$1,852 ($1,790-$1,913)	$238 ($230-$246)	$734 ($672-$795)
Terminal	$10,852 ($10,409-$11,294)	$939 ($905-$972)	$5,573 ($5,123-$6,022)
**Stage II**			
Surgery	$35,162 ($34,364-$35,959)	$2,596 ($2,525-$2,666)	*
Staging	$10,693 ($9,567-$11,818)	$1,368 ($1,244-$1,491)	*
Initial	$4,782 ($4,619-$4,944)	$645 ($619-$660)	$3,684 ($3,520-$3,847)
Continuing	$2,342 ($2,236-$2,447)	$301 ($284-$318)	$1,232 ($1,126-$1,337)
Terminal	$11,908 ($11,359-$12,456)	$1,002 ($963-$1,040)	$7,553 ($6,992-$8,113)
**Stage III**			
Surgery	$36,896 ($36,210-$37,581	$2,599 ($2,542-$2,655)	**
Staging	$8,709 ($7,332-$10,085)	$1,066 ($952-$1,179)	**
Initial	$6,007 ($5,851-$6,162)	$880 ($853-$899)	$4,926 ($4,769-$5,082)
Continuing	$2,826 ($2,688-$2,963)	$373 ($359-$387)	$1,737 ($1,622-$1,851)
Terminal	$11,575 ($11,140-$12,009)	$1,007 ($964-$1,028)	$7,906 ($7,461-$8,350)
**Stage IV**			
Surgery	$38,574 ($37,412-$39,735)	$2,627 ($2,539-$2,714)	**
Staging	$12,911 ($11,905-$13,916)	$1,589 ($1,481-$1,696)	**
Initial	$8,536 ($8,239-$8,832)	$1,295 ($1,194-$1,299)	$7,474 ($7,175-$7,772)
Continuing	$5,991 ($5,730-$6,251)	$931 ($880-$962)	$4,927 ($4,664-$5,189)
Terminal	$13,857 ($13,436-$14,277)	$1,251 ($1,216-$1,285)	$11,721 ($11,295-$12,146)

*Cancer-attributable costs not calculated for the staging and surgery phases.

We further estimated these costs based on specific treatment modalities within each stage. These detailed phase-specific costs by stage and treatment modality are reported in Text A-B S1 File and Tables D-H S1 File.

## Discussion

Our study analyzed treatment utilization and costs among SEER-Medicare patients diagnosed with colon or rectal cancer and identified several racial/ethnic disparities in rates of treatment receipt and relative phase-specific costs. We also estimated these phase-specific costs by stage at diagnosis and treatment subgroup and found that cancer-attributable costs varied widely between subcategories.

### Disparities in treatment receipt and relative cost ratios

After controlling for all other characteristics, we found that Black colon cancer patients were significantly less likely than Whites to receive treatment with surgery, radiation, or chemotherapy. Black rectal cancer patients were also significantly less likely to receive treatment with surgery or chemotherapy. Additionally, when controlling for factors including treatment type, Black colon and rectal cancer patients incurred statistically significantly greater costs in every phase of care, compared to White patients, except for the staging and initial phases of rectal cancer treatment. These trends were observed to a lesser extent when comparing Hispanic and Asian patients with Whites. Significant differences most often revealed Hispanic and Asian patients to have lower rates of treatment utilization and higher surgery costs. Notably, colon and rectal cancer patients in all three racial/ethnic groups had higher costs in the terminal phase than did White patients.

Factors such as stage at diagnosis, educational level, SES, and personal beliefs can contribute to observed racial/ethnic disparities in treatment utilization and cost in the U.S. [35, 36] SES is, for example, known to exacerbate inequalities in treatment receipt; however, the disparities we identified are not attributable to SES, as we controlled for this variable. Compared to Whites, certain racial/ethnic groups have historically been more likely to be diagnosed with stage III-IV colorectal cancer regardless of SES. [11, 14, 37, 38] We therefore controlled for stage at diagnosis as well as SES. Our results suggest that factors independently attributable to race/ethnicity may contribute to care utilization and cost disparities, even after controlling for known potential confounders. No conclusive evidence exists for any sort of racial/ethnic biological or genetic determinant of CRC outcomes. [39–42] On the other hand, cultural and personal beliefs, though difficult to measure, may substantially impact health outcomes. Indeed, studies have shown that even when rates of specialist consultations are similar between Black and White patients diagnosed with advanced stage disease, Black patients are less likely to undergo treatment in the U.S. [43–45]

Patients also may not seek out appropriate care when a language barrier exists. The need to improve language services in healthcare settings is well-established, but the issue is difficult to resolve given the perceived costs and impracticalities of using professional interpreters throughout the course of care; furthermore, there are generally few resources available to physicians to address language barriers in the absence of more favorable alternatives. [46–49] Poor communication between patients and healthcare providers for other reasons may deleteriously affect health outcomes in ways that are difficult to measure. Prior studies have found that Black, Asian, and Hispanic cancer patients are more likely to have lower quality communication as measured by access to clear information on treatment pros and cons and prognosis; time devoted to patient-centered communication and relationship-building between patients and healthcare providers; responsiveness to patient requests; patients’ willingness to ask questions related to their care and condition; and knowledge about who to go to for desired information. [50–54] General mistrust of the healthcare system among Black Americans is also well-established and can impede patients from seeking out needed care. [55–57] It is challenging to overcome these disparities within the American healthcare system.

A greater comorbid disease burden among Black patients may influence observed disparities. Most studies on the subject have found that cancer patients with comorbidity are less likely to receive treatment with curative intent. Physicians may not deem the benefits of such therapy worth the risks of toxicity and of exacerbating a patient’s pre-existing conditions. [58] These findings may validate our results suggesting racial/ethnic disparities in cancer treatment utilization. The causal connection between comorbidity and cost disparity by race/ethnicity is, however, unknown. In order to investigate this connection, a causal inference analysis is needed, which is beyond the scope of our study. [59] More generally, our cost estimates potentially reflect the financial effects of suboptimal disease management. While less likely to receive surgery, radiation, and chemotherapy, certain minority groups also had higher phase and cancer-specific costs even after SES, geographic region, stage of diagnosis, treatment type, and comorbidity were controlled for. Earlier studies have shown that racial/ethnic minority groups are less likely to receive appropriate cancer care at the optimal time. [60–63] Failure to alleviate the disease burden in its early stages or to address accompanying comorbidities can culminate in more intensive and costly end-of-life treatment, with higher rates of emergency visits and hospitalizations. [64–67] Of note, racial/ethnic minorities in the U.S. are also generally less likely to use palliative care and hospice services, compounding high end-of-life expenditures as hospital-based end-of-life care is more likely to be aggressive, and therefore costly, in comparison. [68–71] Our high terminal phase cost estimates for racial/ethnic minorities may reflect these realities. The end-of life period, however, is not the only point at which low treatment utilization may translate to higher cancer-attributable costs. This may occur as early as the initial phase, as our results reveal higher initial and continuing phase costs for Black patients than for Whites. Further research is needed into the specific aspects of care that may be driving these earlier cost trends. More broadly, the influence of patient beliefs, preferences, and provider interactions on treatment decisions and disease management must be better understood in order to reduce disparities in colorectal cancer outcomes.

### Stage and phase-specific cost estimates

Overall, regardless of stage at diagnosis and cancer type, mean monthly total costs were highest in the terminal phase. Costs were lower in the staging phase, followed by the initial phase, and lowest in the continuing phase. Mean monthly total costs in the surgery phase were high, regardless of stage at diagnosis and cancer type. Given that most colorectal cancer patients in our sample received surgery, most treatment-related costs are attributable to the surgery phase rather than the initial phase, especially among patients presenting with stage I disease. Radiation and chemotherapy treatment rates increased as stage at diagnosis increased; therefore, initial phase cost estimates increased in tandem, as patients diagnosed at later stages typically receive these treatments over the course of several months.

Colorectal cancer treatment cost estimates have been previously published, but these rely on SEER-Medicare data prior to 2007. Our estimates use more recent data. [16, 17] Compared to our figures for overall cancer-attributable costs, those reported in prior studies are lower; Brown et al., for example, estimated a mean annual terminal phase cost of $26,200 (or $2,183/month) in 1994 U.S. dollars for stage IV CRC patients, [16] and Lang et al reported a mean annual terminal phase cost of $27,898 (or $2,324/month) in 2006 dollars for stage IV colon cancer patients. [17] In comparison, we estimated a mean monthly cancer-attributable cost of $13,316 in 2019 dollars for stage IV colon cancer patients. Together these results suggest that cancer costs have been rising over time. We also define the initial and terminal phases as six months each, with the initial phase beginning one month after diagnosis, rather than adopting the 12-month definition used in the previous studies. We believe our methods more precisely circumscribe the financially distinct phases of CRC care and, thus, allow us to more accurately determine costs.

### Strengths and limitations

Our study contributes to existing literature by demonstrating the persistence of potential differences in treatment utilization and cancer-attributable costs among racial/ethnic subgroups throughout the course of care. We also provide updated, detailed cost estimates using more recent data, which are a better reflection of current treatment patterns. However, there are several limitations inherent in claims data analyses. Our study was limited to patients over the age of 65 who were diagnosed in a SEER region. Therefore, our results may not be generalizable to younger patients or the entire U.S. population. Our study cohort is limited to those patients who were continuously enrolled in Medicare Parts A and B and who had no HMO for the entire study period. This requirement could potentially bias the analysis by excluding those with noncontinuous Medicare enrollment, or who had their care covered by other forms of insurance. We are unable to determine whether patient-liability costs were paid out-of-pocket or covered by a Medigap program. Since we are not able to determine individual SES from SEER-Medicare, we used an aggregate variable, which many not reflect true SES. However, this method has been determined to be effective for this database. We were not able to determine which treatments received by stage IV patients were palliative. Finally, some costs may have been misclassified. For example, since our study end date was December 31, 2014, patients who died in early 2015 do not have terminal phase costs.

In conclusion, racial/ethnic disparities in CRC treatment receipt persist, with some groups incurring higher care costs during specific phases of care. In addition, the updated cost estimates for CRC care remain substantial and vary widely by phase, cancer site, stage at diagnosis, and treatment modality. These findings are useful for health care professionals seeking to identify potential disparities in care. Our cost estimates may also help to guide future resource allocation and reduce CRC burden.

## Supporting information

S1 File Figure, Text, and Tables. (DOCX)
