# Supplementary material for: Racial/ethnic disparities in colorectal cancer treatment utilization and phase-specific costs, 2000-2014
Source: PLoS One. 2020 Apr 14;15(4):e0231599. doi: 10.1371/journal.pone.0231599 (PMC7156060; doi:10.1371/journal.pone.0231599)
Supplement: S1 File — (DOCX) [file pone.0231599.s001.docx]

**Contents:**

Figure A: Inclusion and exclusion criteria flowchart

Table A: SEER-Medicare claims codes

Table B: Association between total costs and characteristics in the staging and surgery phases: colon cancer

Table C: Association between total costs and characteristics in the staging and surgery phases: rectal cancer

Text A: Specific costs during the staging and surgery phases

Table D: Mean monthly cost estimates by AJCC stage at diagnosis and treatment modality during the staging phase

Table E: Mean monthly cost estimates by AJCC stage at diagnosis during the surgery phase

Text B: Cancer-attributable costs by phase

Table F: Mean monthly cost estimates by AJCC stage at diagnosis and treatment modality during the initial phase

Table G: Mean monthly cost estimates by AJCC stage at diagnosis and treatment modality during the continuing phase

Table H: Mean monthly cost estimates by AJCC stage at diagnosis and treatment modality during the terminal phase

**Figure A: Inclusion and exclusion criteria flowchart**

**
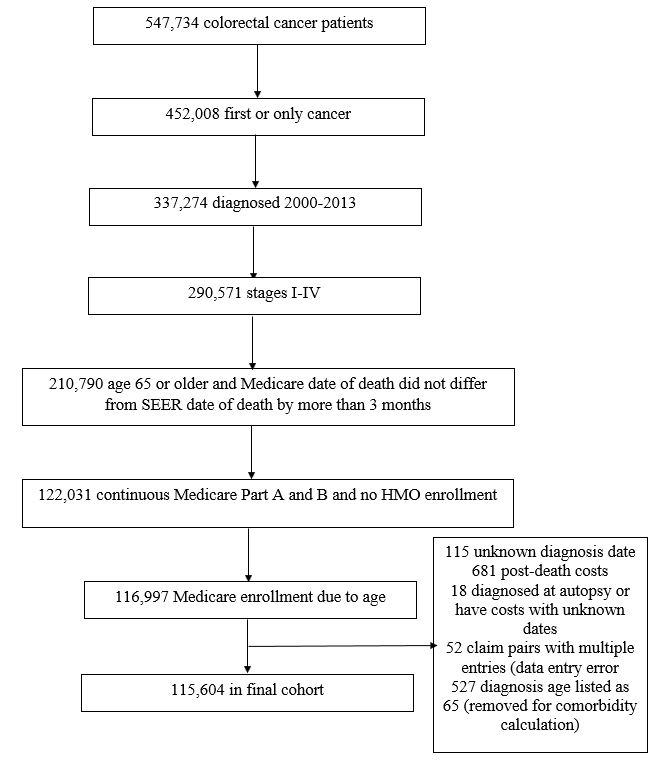
**

**Table A: SEER-Medicare claims codes**

| Variable | Source | Codes |
| --- | --- | --- |
| Surgery | inpatient, outpatient, physician, hospice, home health, or durable medical equipment claims | ICD-9-CM: 45.31-45.34, 45.41-45.43, 45.49-45.52, 45.60, 45.70-45.83, 45.90-45.95, 48.61-48.65, 48.35, 48.40-48.43, 48.49-48.51, 48.52, 48.59,  CPT: 44140, 44141, 44143-44147, 44150-44160, 44204-44208, 44210-44212, 45110-45114, 45119, 45123, 45126, 45395, 45397, 45160, 45170 |
| Radiation | inpatient, outpatient, physician, hospice, home health, or durable medical equipment claims | ICD-9-CM: V58.0, V66.1 V67.1, 92.21-92.29  HCPCS: code S8049  CPT: 77401-77499, 77750-77799, G0256, G0261  Revenue Center: 0330, 0333, 0339 |
| Chemotherapy | inpatient, outpatient, physician, hospice, home health, or durable medical equipment claims | ICD-9-CM: V58.1, V58.11, 99.25  HCPCS: C1166, C1167, C1178, C9110, C9205, C9207, C9213-C9216, C9411, C9414-C9419, C9420-C9438, G0355, G0356, G0359-G0362, J7150, J8500-J8799, J8999-J9999, Q0083-Q0085, S9325-S9329, S9330-S9379, or S9494-S9497  CPT: 96400-96546  Revenue Center 0331, 0332, 0335 |

**Table A:** **Association Between** **Total Costs and Characteristics in the Staging and Surgery Phases: Colon Cancer**

|  | **Staging Phase** | | | **Surgery Phase** | | |
| --- | --- | --- | --- | --- | --- | --- |
| **Characteristic** | **Relative cost** | **95% CI** | **P-value** | **Relative cost** | **95% CI** | **P-value** |
| Race/Ethnicity ref=White |  |  |  |  |  |  |
| Black | 1.19 | 1.02-1.40 | 0.03* | 1.11 | 1.09-1.13 | <0.0001* |
| Hispanic | 1.03 | 0.83-1.27 | 0.79 | 1.02 | 1.00-1.05 | 0.046* |
| Asian | 1.20 | 0.95-1.52 | 0.12 | 1.08 | 1.06-1.11 | <0.0001* |
| Other | 0.83 | 0.51-1.36 | 0.46 | 0.93 | 0.87-1.00 | 0.049* |
| Sex ref=Male |  |  |  |  |  |  |
| Female | 1.26 | 1.14-1.39 | <0.0001* | 1.00 | 0.99-1.01 | 0.48 |
| Age at diagnosis ref=66-69 |  |  |  |  |  |  |
| 70-74 | 1.12 | 0.95-1.31 | 0.17 | 1.01 | 0.99-1.02 | 0.29 |
| 75-79 | 1.15 | 0.98-1.34 | 0.09 | 1.04 | 1.03-1.06 | <0.0001* |
| 80-84 | 1.39 | 1.18-1.64 | <0.0001* | 1.07 | 1.06-1.09 | <0.0001* |
| 85+ | 1.90 | 1.61-2.24 | <0.0001* | 1.16 | 1.14-1.18 | <0.0001* |
| Marital Status ref=Unmarried |  |  |  |  |  |  |
| Married | 1.10 | 0.99-1.23 | 0.08 | 1.06 | 1.05-1.08 | <0.0001* |
| Unknown | 0.78 | 0.64-0.94 | 0.009* | 1.06 | 1.03-1.09 | <0.0001* |
| SES ref=0 |  |  |  |  |  |  |
| 1 | 0.93 | 0.80-1.07 | 0.31 | 1.00 | 0.98-1.01 | 0.67 |
| 2 | 0.97 | 0.83-1.12 | 0.66 | 0.98 | 0.97-1.00 | 0.02* |
| 3 | 0.95 | 0.82-1.11 | 0.55 | 0.98 | 0.96-0.99 | 0.004* |
| 4 highest | 1.01 | 0.86-1.18 | 0.91 | 0.96 | 0.95-0.98 | <0.0001* |
| SEER Region ref=Northeast |  |  |  |  |  |  |
| South | 1.06 | 0.92-1.23 | 0.40 | 0.83 | 0.82-0.84 | <0.0001* |
| Midwest | 0.80 | 0.68-0.95 | 0.009* | 0.84 | 0.83-0.85 | <0.0001* |
| West/Hawaii | 0.94 | 0.83-1.06 | 0.32 | 0.98 | 0.97-0.99 | 0.001* |
| Residence ref=Metro/Urban |  |  |  |  |  |  |
| Less Urban/Rural | 1.02 | 0.87-1.20 | 0.81 | 0.91 | 0.89-0.92 | <0.0001 |
| Year of Dx ref=2000-2004 |  |  |  |  |  |  |
| 2005-2009 | 1.09 | 0.97-1.22 | 0.14 | 1.00 | 0.99-1.01 | 0.77 |
| 2010-2013 | 1.33 | 1.18-1.50 | <0.0001* | 0.88 | 0.87-0.89 | <0.0001* |
| AJCC Stage ref=I |  |  |  |  |  |  |
| II | 1.15 | 0.99-1.34 | 0.07 | 1.25 | 1.24-1.26 | <0.0001* |
| III | 0.74 | 0.61-0.90 | 0.003* | 1.37 | 1.35-1.39 | <0.0001* |
| IV | 1.65 | 1.44-1.90 | <0.0001* | 1.49 | 1.46-1.52 | <0.0001* |
| Charlson Score ref=0 |  |  |  |  |  |  |
| 1 | 1.35 | 1.20-1.51 | <0.0001* | 1.11 | 1.1-1.12 | <0.0001* |
| 2+ | 2.13 | 1.9-2.38 | <0.0001* | 1.28 | 1.26-1.29 | <0.0001* |
| Treatment ref=BSC** |  |  |  |  |  |  |
| Radiation | 1.52 | 1.02-2.26 | 0.04* | 1.09 | 1.04-1.16 | 0.001* |
| Chemotherapy | 1.44 | 1.25-1.66 | <0.0001* | 0.97 | 0.96-0.98 | <0.0001* |
| Chemoradiation | 1.66 | 1.3-2.12 | 0.0001* | 1.01 | 0.97-1.04 | 0.73 |

*P-value significant at <0.05 for multiple linear regression using log transformation of cost

**Reference=Surgery only in surgery phase regression

**Table B: Association Between** **Total Costs and Characteristics in the Staging and Surgery Phases: Rectal Cancer**

|  | **Staging Phase** | | | **Surgery Phase** | | |
| --- | --- | --- | --- | --- | --- | --- |
| **Characteristic** | **Relative cost** | **95% CI** | **P-value** | **Relative cost** | **95% CI** | **P-value** |
| Race/Ethnicity ref=White |  |  |  |  |  |  |
| Black | 1.16 | 0.99-1.36 | 0.07 | 1.08 | 1.03-1.14 | 0.003* |
| Hispanic | 1.07 | 0.9-1.28 | 0.45 | 1.05 | 0.99-1.11 | 0.08 |
| Asian | 0.90 | 0.74-1.09 | 0.28 | 1.04 | 0.99-1.1 | 0.16 |
| Other | 0.84 | 0.55-1.26 | 0.40 | 0.85 | 0.71-1.01 | 0.07 |
| Sex ref=Male |  |  |  |  |  |  |
| Female | 1.09 | 1.00-1.19 | 0.047* | 0.97 | 0.94-0.99 | 0.009* |
| Age at diagnosis ref=66-69 |  |  |  |  |  |  |
| 70-74 | 0.92 | 0.80-1.05 | 0.21 | 0.97 | 0.94-1.00 | 0.07 |
| 75-79 | 0.94 | 0.82-1.07 | 0.34 | 0.99 | 0.95-1.03 | 0.55 |
| 80-84 | 1.06 | 0.92-1.22 | 0.40 | 1.00 | 0.96-1.04 | 0.96 |
| 85+ | 1.26 | 1.09-1.45 | 0.002* | 1.02 | 0.98-1.07 | 0.37 |
| Marital Status ref=Unmarried |  |  |  |  |  |  |
| Married | 1.21 | 1.10-1.32 | <0.0001* | 1.05 | 1.02-1.08 | 0.0003* |
| Unknown | 1.02 | 0.85-1.22 | 0.86 | 1.04 | 0.98-1.11 | 0.15 |
| SES ref=0 |  |  |  |  |  |  |
| 1 | 0.98 | 0.86-1.11 | 0.74 | 1.00 | 0.96-1.04 | 0.93 |
| 2 | 0.89 | 0.78-1.02 | 0.09 | 0.97 | 0.94-1.01 | 0.16 |
| 3 | 0.85 | 0.74-0.97 | 0.02* | 1.01 | 0.97-1.04 | 0.78 |
| 4 highest | 0.93 | 0.80-1.07 | 0.29 | 0.98 | 0.94-1.02 | 0.33 |
| SEER Region ref=Northeast |  |  |  |  |  |  |
| South | 1.00 | 0.88-1.14 | 0.95 | 0.84 | 0.81-0.87 | <0.0001* |
| Midwest | 1.00 | 0.86-1.16 | 1.00 | 0.84 | 0.81-0.88 | <0.0001* |
| West/Hawaii | 0.99 | 0.89-1.11 | 0.89 | 0.97 | 0.94-1.00 | 0.08 |
| Residence ref=Metro/Urban |  |  |  |  |  |  |
| Less Urban/Rural | 0.94 | 0.82-1.09 | 0.42 | 0.92 | 0.89-0.96 | <0.0001* |
| Year of Dx ref=2000-2004 |  |  |  |  |  |  |
| 2005-2009 | 1.05 | 0.95-1.17 | 0.36 | 1.16 | 1.13-1.2 | <0.0001* |
| 2010-2013 | 1.07 | 0.96-1.19 | 0.22 | 1.28 | 1.24-1.32 | <0.0001* |
| AJCC Stage ref=I |  |  |  |  |  |  |
| II | 1.27 | 1.13-1.44 | <0.0001* | 1.53 | 1.48-1.58 | <0.0001* |
| III | 0.99 | 0.86-1.13 | 0.86 | 1.68 | 1.63-1.74 | <0.0001* |
| IV | 1.83 | 1.62-2.08 | <0.0001* | 1.78 | 1.69-1.86 | <0.0001* |
| Charlson Score ref=0 |  |  |  |  |  |  |
| 1 | 1.38 | 1.24-1.53 | <0.0001* | 1.11 | 1.08-1.14 | <0.0001* |
| 2+ | 1.85 | 1.67-2.05 | <0.0001* | 1.27 | 1.23-1.31 | <0.0001* |
| Treatment ref=BSC |  |  |  |  |  |  |
| Radiation | 1.25 | 1.11-1.41 | 0.0003* | 0.84 | 0.8-0.87 | <0.0001* |
| Chemotherapy | 1.26 | 1.06-1.50 | 0.009* | 0.96 | 0.92-1.00 | 0.08 |
| Chemoradiation | 1.27 | 1.13-1.42 | <0.0001* | 0.92 | 0.89-0.95 | <0.0001* |

*P-value significant at <0.05 for multiple linear regression using log transformation of cost

**Reference=Surgery only in surgery phase regression

**Text A: Specific costs during the staging and surgery phases**

Among colon cancer patients, mean (95% CI) total staging costs ranged from $6,208 ($4,295-$8,121) for stage III patients receiving best supportive care to $15,590 ($14,005-$17,176) for stage IV patients receiving best supportive care. The mean (95% CI) total costs during the surgery phase ranged from $32,782 ($32,000-$33,142) for stage I patients to $41,818 ($41,266-$42,371) for stage IV patients.

Among rectal cancer patients, mean (95% CI) total staging costs ranged from $7,248 ($6,348-$8,150) for stage I patients receiving best supportive care to $16,622 ($13,446-19,798) for stage IV patients receiving best supportive care. Mean (95% CI) total costs during the surgery phase ranged from $27,284 ($26,583-$27,984) for stage I patients to $38,574 ($37,412-$39,735) for stage IV patients.

In this cohort, 897 (1.2%) colon cancer patients and 2,342 (13.0%) rectal cancer patients received chemotherapy before surgery. The median (25^th^, 75^th^ percentile) time for the presugery phase was 3.1 months (1.0, 6.3) for colon cancer surgical patients with neoadjuvant chemotherapy and 4.5 months (3.9, 5.2) for rectal cancer surgical patients with neoadjuvant chemotherapy. The mean (95% CI) monthly total costs were $9,621 ($9,093-$10,149) and $8,096 ($7,890-$8,303) for colon and rectal cancer patients, respectively. The median (25^th^, 75^th^ percentile) time for the presugery phase was 0.8 months (0.4, 1.2) for colon cancer surgical patients without neoadjuvant chemotherapy and 0.9 months (0.5, 1.6) for rectal cancer surgical patients without neoadjuvant chemotherapy. The mean (95% CI) monthly total costs were $4,482 ($4,385-$4,579) and $5,293 ($5,068-$5,518) for colon and rectal cancer patients, respectively.

**Table D: Mean monthly cost estimates by AJCC stage at diagnosis and treatment modality during the staging phase**

|  | N | Total Cost  (95% CI) | Patient-liability cost  (95% CI) |
| --- | --- | --- | --- |
| *Colon* |  |  |  |
| **Stage I** | 1,691 |  |  |
| Best Supportive Care | 1,633 (96.5%) | $7,672 ($6,929-$8,415) | $941 ($865-$1,016) |
| **Stage II** | 739 |  |  |
| Best Supportive Care | 637 (86.2%) | $8,968 ($7,496-$10,440) | $724 ($614-$835) |
| **Stage III** | 431 |  |  |
| Best Supportive Care | 329 (76.3%) | $6,208 ($4,295-$8,121) | $542 ($391-$692) |
| Chemotherapy | 87 (20.1%) | $10,630 ($5,668-$15,592) | $744 ($555-$934) |
| **Stage IV** | 1,604 |  |  |
| Best Supportive Care | 485 (30.3%) | $15,590 ($14,005-$17,176) | $1,561 ($1,399-$1,723) |
| Chemotherapy | 920 (57.4%) | $12,528 ($11,665-$13,390) | $1,559 ($1,481-$1,636) |
| Chemoradiation | 168 (10.5%) | $13,820 ($11,658-$15,983) | $1,628 ($1,452-$1,804) |
| *Rectal* |  |  |  |
| **Stage I** | 1,603 |  |  |
| Best Supportive Care | 1,028 (64.2%) | $7,248 ($6,348-$8,150) | $942 ($848-$1,037) |
| Radiation | 258 (16.1%) | $9,569 ($7,941-$11,197) | $1,353 ($1,142-$1,565) |
| Chemoradiation | 276 (17.2%) | $7,873 ($6,526-$9,220) | $1,283 ($1,104-$1,462) |
| **Stage II** | 803 |  |  |
| Best Supportive Care | 190 (23.7%) | $8,911 ($6,697-$11,125) | $1,005 ($760-$1,250) |
| Radiation | 283 (35.2%) | $11,404 ($9,258-$13,549) | $1,528 ($1,262-$1,794) |
| Chemoradiation | 304 (37.7%) | $10,658 ($9,005-$12,311) | $1,433 ($1,275-$1,592) |
| **Stage III** | 644 |  |  |
| Best Supportive Care | 142 (22.1%) | $9,277 ($4,744-$13,810) | $718 ($454-$981) |
| Radiation | 184 (28.5%) | $910,087 ($7,693-$12,482) | $1,361 ($1,075-$1,646) |
| Chemoradiation | 289 (44.9%) | $7,692 ($6,264-$9,120) | $1,070 ($959-$1,180) |
| **Stage IV** | 862 |  |  |
| Best Supportive Care | 154 (16.6%) | $16,622 ($13,446-19,798) | $1,900 ($1,470-$2,331) |
| Chemotherapy | 283 (30.5%) | $12,492 ($10,964-$14,019) | $1,570 ($1,420-$1,720) |
| Chemoradiation | 380 (41.0%) | $11,360 ($10,002-$12,718) | $1,496 ($1,360-$1,632) |

**Table E: Mean monthly cost estimates by AJCC stage at diagnosis during the surgery phase**

|  |  |  |  |
| --- | --- | --- | --- |
|  | N | Total Cost (95% CI) | Patient-liability cost (95% CI) |
| *Colon* |  |  |  |
| **Stage I** | 17,109 | $32,782 ($32,000-$33,142) | $2,212 ($2,190-$2,240) |
| **Stage II** | 23,883 | $38,681 ($38,364-$38,999) | $2,464 ($2,440-$2,490) |
| **Stage III** | 18,505 | $39,961 ($39,607-$40,316) | $2,504 ($2,470-$2,530) |
| **Stage IV** | 6,341 | $41,818 ($41,266-$42,371) | $2,631 ($2,580-$2,680) |
| *Rectal* |  |  |  |
| **Stage I** | 5,855 | $27,284 ($26,583-$27,984) | $2,160 ($2,115-$2,204) |
| **Stage II** | 4,627 | $35,162 ($34,364-$35,959) | $2,596 ($2,525-$2,666) |
| **Stage III** | 4,887 | $36,896 ($36,210-$37,581 | $2,599 ($2,542-$2,655) |
| **Stage IV** | 1,565 | $38,574 ($37,412-$39,735) | $2,627 ($2,539-$2,714) |

**Text B: Cancer-attributable costs of treatment by phase**

Among colon cancer patients in the initial phase, those who received surgery and chemotherapy had the highest mean (95% CI) cancer attributable costs in stages II-IV, ranging from $3,810 ($3,632-$3,987) for stage II patients to $8,092 ($7,881-$8,303) for stage IV patients. Among rectal cancer patients, mean (95% CI) monthly cancer-attributable costs ranged from $863 ($637-$1,089) for stage I patients receiving best supportive care to $9,876 ($9,028-$10,724) for stage IV patients receiving chemoradiation.

Among colon cancer patients in the continuing phase, the highest mean (95% CI) monthly cancer-attributable costs for stage I was $718 ($583-$852) for those receiving best supportive care, for stage II was $1,024 ($893-$1,154) for those receiving surgery and chemotherapy, for stage III was $1,430 ($1,362-$1,499) for those receiving surgery and chemotherapy, and for stage IV was $5,102 ($4,756-$5,449) for those receiving surgery and chemoradiation. Within each rectal cancer stage, the highest mean (95% CI) monthly cancer attributable costs were $874 ($666-$1,081) for stage I patients receiving best supportive care, $1,627 ($849-$2,405) for stage II patients receiving best supportive care, $1,701 ($1,290-$2,112) for stage III patients receiving surgery and chemoradiation, and $6,430 ($5,527-$7,333) for stage IV patients receiving chemoradiation.

Mean (95% CI) monthly cancer-attributable costs among colon cancer patients in the terminal phase ranged from $5,307 ($4,928-$5,687) for stage I surgical to $18,200 ($17,485-$18,914) for stage IV surgical patients. The costs for stages II and III were highest for surgical patients, at $7,027 ($6,696-$7,358) and $9,936 ($9,484-$10,387) for stage II and III, respectively. Among rectal cancer patients, mean (95% CI) monthly cancer-attributable costs ranged from $4,967 ($4,192-$5,742) for stage I patients who received best supportive care to $15,671 ($14,109-$17,233) for stage IV surgical patients. Surgical patients had the highest costs in stages I-III, at $5,889 ($5,183-$6,596), $8,534 ($7,416-$9,652) and $9,503 ($8,480-$10,526), for stage I, II, and II, respectively.

**Table F.** **Mean monthly cost estimates by AJCC stage at diagnosis and treatment modality during the initial phase**

|  |  |  |  |  |
| --- | --- | --- | --- | --- |
|  | N (%) | Total Cost (95% CI) | Patient-liability cost (95% CI) | Cancer-attributable cost (95% CI) |
| *Colon* |  |  |  |  |
| **Stage I** | 18,667 |  |  |  |
| Best supportive care | 1,593 (8.5%) | $2,328 ($2,092-$2,563) | $233 ($212-$255) | $1,212 ($976-$1,447) |
| Surgery | 16,534 (88.6%) | $1,909 ($1,845-$1,972) | $225 ($215-$232) | $798 ($735-$861) |
| **Stage II** | 24,410 |  |  |  |
| Best supportive care | 627 (2.5%) | $2,531 ($2,093-$2,969) | $224 ($185-$264) | $1,417 ($979-$1,856) |
| Surgery | 20,044 (82.1%) | $2,120 ($2,059-$2,181) | $255 ($247-$263) | $990 ($928-$1,051) |
| Surgery and Chemotherapy | 3,193 (13.1%) | $4,890 ($4,714-$5,066) | $780 ($755-$805) | $3,810 ($3,632-$3,987) |
| **Stage III** | 18,614 |  |  |  |
| Best supportive care | 323 (1.7%) | $2,565 ($2,054-$3,075) | $237 ($184-$289) | $1,458 ($948-$1,969) |
| Surgery | 8,346 (44.8%) | $3,165 ($3,043-$3,286) | $354 ($338-$370) | $2,011 ($1,889-$2,132) |
| Surgery and Chemotherapy | 9,480 (50.9%) | $6,583 ($6,474-$6,691) | $1,076 ($1,059-$1,094) | $5,518 ($5,409-$5,628) |
| **Stage IV** | 7,475 |  |  |  |
| Best supportive care | 406 (5.4%) | $4,959 ($4,268-$5,650) | $441 ($347-$535) | $3,826 ($3,133-$4,518) |
| Surgery | 1,384 (18.5%) | $3,442 ($3,146-$3,737) | $352 ($312-$393) | $2,295 ($1,999-$2,591) |
| Surgery and Chemotherapy | 3,749 (50.2%) | $9,149 ($8,940-$9,358) | $1,478 ($1,444-$1,513) | $8,092 ($7,881-$8,303) |
| *Rectal* |  |  |  |  |
| **Stage I** | 7,409 |  |  |  |
| Best supportive care | 1,014 (13.7%) | $1,970 ($1,744-$2,196) | $253 ($219-$281) | $863 ($637-$1,089) |
| Surgery | 4,516 (61.0%) | $2,217 ($2,087-$2,347) | $253 ($233-$270) | $1,099 ($969-$1,230) |
| **Stage II** | 5,408 |  |  |  |
| Best supportive care | 192 (3.5%) | $3,329 ($2,572-$4,0837 | $357 ($257-$458) | $2,194 ($1,438-$2,950) |
| Surgery | 2,156 (39.9%) | $2,625 ($2,418-$2,832) | $293 ($267-$320) | $1,494 ($1,287-$1,702) |
| Surgery and Radiation | 730 (13.5%) | $5,181 ($4,721-$5,642) | $692 ($641-$753) | $4,114 ($3,653-$4,576) |
| Surgery and Chemoradiation | 1,424 (26.3%) | $6,001 ($5,682-$6,320) | $878 ($840-$915) | $4,933 ($4,613-$5,253) |
| **Stage III** | 5,459 |  |  |  |
| Best supportive care | 138 (2.5%) | $3,217 ($2,118-$4,317) | $380 ($246-$514) | $2,145 ($1,045-$32,44) |
| Surgery | 1,393 (25.4%) | $3,739 ($3,397-$4,080) | $447 ($391-$499) | $2,587 ($2,245-$2,929) |
| Surgery and Radiation | 590 (11.0%) | $5,926 ($5,481-$6,371) | $782 ($725-$840) | $4,866 ($4,419-$5,314) |
| Surgery and Chemotherapy | 976 (17.9%) | $6,537 ($6,208-$6,865) | $1,025 ($977-$1,074) | $5,469 ($5,137-$5,801) |
| Surgery and Chemoradiation | 1,875 (34.4%) | $6,758 ($6,536-$6,980) | $1,066 ($1,033-$1,098) | $5,707 ($5,483-$5,930) |
| **Stage IV** | 2,367 |  |  |  |
| Best supportive care | 136 (5.8%) | $4,615 ($3,444-$5,786) | $391 ($261-$521) | $3,500 ($2,329-$4,670) |
| Chemoradiation | 267 (11.3%) | $10,930 ($10,083-$11,777) | $1,703 ($1,603-$1,802) | $9,876 ($9,028-$10,724) |
| Surgery and Chemotherapy | 607 (25.6%) | $8,966 ($8,447-$9,484) | $1,425 ($1,335-$1,514) | $7,908 ($7,386-$8,431) |
| Surgery and Chemoradiation | 578 (24.4%) | $8,625 ($8,113-$9,138) | $1,253 ($1,177-$1,329) | $7,598 ($7,083-$8,113) |

**Table G. Mean monthly cost estimates by AJCC stage at diagnosis and treatment modality during the continuing phase**

|  | N (%) | Total Cost (95% CI) | Patient-liability cost  (95% CI) | Cancer-attributable cost (95% CI) |
| --- | --- | --- | --- | --- |
| *Colon* |  |  |  |  |
| **Stage I** | 17,978 |  |  |  |
| Best supportive care | 1,442 (8.0%) | $1,842 ($1,708-$1,977) | $222 ($204-$241) | $718 ($583-$852) |
| Surgery | 16,031 (89.2%) | $1,651 ($1,609-$1,693) | $200 ($215-$225) | $528 ($486-$570) |
| **Stage II** | 23,227 |  |  |  |
| Best supportive care | 579 (2.5%) | $1,708 ($1,462-$1,954) | $191 ($165-$218) | $591 ($344-$838) |
| Surgery | 19,071 (82.2%) | $1,746 ($1,708-$1,784) | $231 ($226-$236) | $603 ($565-$642) |
| Surgery and Chemotherapy | 3,100 (13.4%) | $2,115 ($1,984-$2,246) | $302 ($242-$319) | $1,024 ($893-$1,154) |
| **Stage III** | 16,892 |  |  |  |
| Best supportive care | 289 (1.7%) | $1,706 ($1,369-$2,042) | $175 ($141-$209) | $592 ($256-$928) |
| Surgery | 7,278 (43.1%) | $2,081 ($2,010-$2,152) | $270 ($257-$281) | $913 ($842-$985) |
| Surgery and Chemotherapy | 8,923 (52.8%) | $2,506 ($2,437-$2,574) | $376 ($366-$388) | $1,430 ($1,362-$1,499) |
| **Stage IV** | 5,193 |  |  |  |
| Best supportive care | 181 (3.5%) | $2,803 ($2,252-$3,354) | $248 ($176-$320) | $1,660 ($1,111-$2,210) |
| Surgery | 812 (15.6%) | $2,352 ($2,085-$2,619) | $254 ($221-$285) | $1,197 ($929-$1,465) |
| Surgery and Chemotherapy | 2,821 (54.2%) | $6,076 ($5,869-$6,283) | $1,009 ($977-$1,042) | $5,014 ($4,806-$5,222) |
| Surgery and Chemoradiation | 685 (13.2%) | $6,149 ($5,804-$6,494) | $1,017 ($959-$1,073) | $5,102 ($4,756-$5,449) |
| *Rectal* |  |  |  |  |
| **Stage I** | 7,035 |  |  |  |
| Best supportive care | 922 (13.1%) | $1,992 ($1,784-$2,200) | $228 ($210-$247) | $874 ($666-$1,081) |
| Surgery | 4,336 (61.6%) | $1,688 ($1,622-$1,755) | $221 ($212-$230) | $559 ($493-$625) |
| **Stage II** | 5,054 |  |  |  |
| Best supportive care | 164 (3.2%) | $2,771 ($1,994-$3,549) | $349 ($167-$530) | $1,627 ($849-$2,405) |
| Surgery | 2,043 (40.4%) | $1,984 ($1,837-$2,132) | $249 ($233-$265) | $837 ($690-$985) |
| Surgery and Radiation | 675 (13.4%) | $2,301 ($2,060-$2,542) | $287 ($258-$317) | $1,225 ($984-$1,467) |
| Surgery and Chemoradiation | 1,353 (26.7%) | $2,198 ($2,041-$2,356) | $298 ($277-$318) | $1,123 ($965-$1,281) |
| **Stage III** | 4,970 |  |  |  |
| Best supportive care | 124 (2.5%) | $2,340 ($1,773-$2,906) | $274 ($200-$346) | $1,256 ($685-$1,828) |
| Surgery | 1,212 (24.4%) | $2,529 ($2,303-$2,755) | $321 ($290-$353) | $1,368 ($1,140-$1,595) |
| Surgery and Radiation | 536 (10.8%) | $2,770 ($2,360-$3,179) | $334 ($300-$369) | $1,701 ($1,290-$2,112) |
| Surgery and Chemotherapy | 924 (18.6%) | $2,658 ($2,462-$2,854) | $396 ($363-$425) | $1,581 ($1,384-$1,779) |
| Surgery and Chemoradiation | 1,747 (35.2%) | $2,944 ($2,739-$3,149) | $406 ($384-$429) | $1,884 ($1,678-$2,090) |
| **Stage IV** | 1,682 |  |  |  |
| Best supportive care | 56 (3.3%) | $2,729 ($1,668-$3,790) | $168 ($62-$274) | $1,606 ($547-$2,665) |
| Chemoradiation | 248 (14.7%) | $7,495 ($6,596-$8,394) | $1,157 ($1,049-$1,266) | $6,430 ($5,527-$7,333) |
| Surgery and Chemotherapy | 480 (28.5%) | $6,822 ($6,378-$7,266) | $1,144 ($1,066-$1,221) | $5,759 ($5,312-$6,206) |
| Surgery and Chemoradiation | 469 (27.9%) | $6,070 ($5,610-$6529) | $945 ($875-$1,015) | $5,038 ($4,576-$5,499) |

**Table H. Mean monthly cost estimates by AJCC stage at diagnosis and treatment modality during the terminal phase**

|  |  |  |  |  |
| --- | --- | --- | --- | --- |
|  | N (%) | Total Cost (95% CI) | Patient-liability cost (95% CI) | Cancer-attributable cost (95% CI) |
| *Colon* |  |  |  |  |
| **Stage I** | 10,687 |  |  |  |
| Best supportive care | 1,421 (13.3%) | $10,485 ($9,810-$11,159) | $858 ($807-$910) | $5,491 ($4,803-$6,178) |
| Surgery | 8,882 (83.1%) | $11,500 ($11,123-$11,877) | $905 ($881-$929) | $5,307 ($4,928-$5,687) |
| **Stage II** | 15,579 |  |  |  |
| Best supportive care | 563 (3.6%) | $9,933 ($9,000-$10,866) | $813 ($725-$900) | $5,581 ($4,586-$6,576) |
| Surgery | 12,911 (82.9%) | $12,149 ($11,823-$12,476) | $926 ($895-$947) | $7,027 ($6,696-$7,358) |
| Surgery and Chemotherapy | 1,660 (10.7%) | $10,321 ($8,438-$12,283) | $944 ($891-$987) | $5,898 ($4,011-$7,785) |
| **Stage III** | 14,415 |  |  |  |
| Best supportive care | 308 (2.1%) | $9,652 ($8,378-$10,925) | $739 ($650-$828) | $5,684 ($4,396-$6,973) |
| Surgery | 8,024 (55.7%) | $13,674 ($13,226-$14,122) | $966 ($939-$994) | $9,936 ($9,484-$10,387) |
| Surgery and Chemotherapy | 5,677 (39.4%) | $10,357 ($10,123-$10,590) | $1,029 ($1,005-$1,053) | $6,733 ($6,487-$6,980) |
| **Stage IV** | 14,208 |  |  |  |
| Best supportive care | 3,316 (22.7%) | $15,298 ($14,835-$15,761) | $1,156 ($1,125-$1,188) | $12,591 ($12,128-$13,054) |
| Surgery | 3,734 (26.7%) | $20,436 ($19,723-$21,150) | $1,159 ($1,120-$1,182) | $18,200 ($17,485-$18,914) |
| Surgery and Chemotherapy | 4,331 (31.2%) | $12,879 ($12,536-$13,223) | $1,302 ($1,273-$1,331) | $10,996 ($10,648-$11,343) |
| *Rectal* |  |  |  |  |
| **Stage I** | 4,774 |  |  |  |
| Best supportive care | 794 (16.6%) | $9,711 ($8,942-$10,479) | $851 ($756-$927) | $4,967 ($4,192-$5,742) |
| Surgery | 2,721 (57.0%) | $11,562 (10,865-$12,260) | $942 ($896-$988) | $5,889 ($5,183-$6,596) |
| **Stage II** | 3,811 |  |  |  |
| Best supportive care | 232 (6.1%) | $10,638 ($9,161-$12,114) | $887 ($764-$1,010) | $7,175 ($5,640-$8,710) |
| Surgery | 1,634 (42.9%) | $13,109 ($12,008-$14,209) | $986 ($921-$1,051) | $8,534 ($7,416-$9,652) |
| Surgery and Radiation | 429 (11.3%) | $10,752 ($9,685-$11,818) | $956 ($848-$1,063) | $6,625 ($5,528-$7,723) |
| Surgery and Chemoradiation | 823 (21.6%) | $10,772 ($9,929-$11,616) | $980 ($903-$1,057) | $6,122 ($5,243-$7,001) |
| **Stage III** | 4,106 |  |  |  |
| Best supportive care | 151 (3.7%) | $9,823 ($8,066-$11,579) | $829 ($672-$987) | $6,118 ($4,310-$7,926) |
| Surgery | 1,368 (33.3%) | $13,234 ($12,213-$14,254) | $997 ($933-$1,062) | $9,503 ($8,480-$10,526) |
| Surgery and Chemotherapy | 598 (14.6%) | $10,113 ($9,355-$10,871) | $1,003 ($929-$1,077) | $6,328 ($5,525-$7,131) |
| Surgery and Chemoradiation | 1,215 (29.6%) | $10,920 ($10,307-$11,534) | $1,014 ($963-$1,066) | $7,312 ($6,669-$7,955) |
| **Stage IV** | 3,934 |  |  |  |
| Best supportive care | 7357 (18.7%) | $15,209 ($14,321-$16,098) | $1,115 ($1,048-$1,181) | $12,854 ($11,944-$13,763) |
| Chemotherapy | 436 (11.1%) | $13,113 ($12,201-$14,025) | $1,480 ($1,337-$1,623) | $11,341 ($10,421-$12,262) |
| Chemoradiation | 490 (11.9%) | $11,867 ($11,110-$12,625) | $1,413 ($1,326-$1,501) | 9,647 ($8,873-$10,421) |
| Surgery | 627 (16.0%) | $17,946 ($16,376-$19,516) | $1,130 ($1,030-$1,230) | $15,671 ($14,109-$17,233) |
| Surgery and Chemotherapy | 701 (17.9%) | $11,741 ($10,775-$12,706) | $1,187 ($1,117-$1,256) | $9,703 ($8,716-$10,690) |
| Surgery and Chemoradiation | 597 (15.2%) | $12,470 ($11,426-$13,514) | $1,284 ($1,202-$1,367) | $10,521 ($9,456-$11,587) |
